# Assessing Concordance of Drug-Induced Transcriptional Response in Rodent Liver and Cultured Hepatocytes

**DOI:** 10.1371/journal.pcbi.1004847

**Published:** 2016-03-30

**Authors:** Jeffrey J. Sutherland, Robert A. Jolly, Keith M. Goldstein, James L. Stevens

**Affiliations:** Lilly Research Laboratories, Eli Lilly and Company, Indianapolis, Indiana, United States of America; National University of Singapore, SINGAPORE

## Abstract

The effect of drugs, disease and other perturbations on mRNA levels are studied using gene expression microarrays or RNA-seq, with the goal of understanding molecular effects arising from the perturbation. Previous comparisons of reproducibility across laboratories have been limited in scale and focused on a single model. The use of model systems, such as cultured primary cells or cancer cell lines, assumes that mechanistic insights derived from the models would have been observed via *in vivo* studies. We examined the concordance of compound-induced transcriptional changes using data from several sources: rat liver and rat primary hepatocytes (RPH) from Drug Matrix (DM) and open TG-GATEs (TG), human primary hepatocytes (HPH) from TG, and mouse liver / HepG2 results from the Gene Expression Omnibus (GEO) repository. Gene expression changes for treatments were normalized to controls and analyzed with three methods: 1) gene level for 9071 high expression genes in rat liver, 2) gene set analysis (GSA) using canonical pathways and gene ontology sets, 3) weighted gene co-expression network analysis (WGCNA). Co-expression networks performed better than genes or GSA when comparing treatment effects within rat liver and rat vs. mouse liver. Genes and modules performed similarly at Connectivity Map-style analyses, where success at identifying similar treatments among a collection of reference profiles is the goal. Comparisons between rat liver and RPH, and those between RPH, HPH and HepG2 cells reveal lower concordance for all methods. We observe that the baseline state of untreated cultured cells relative to untreated rat liver shows striking similarity with toxicant-exposed cells *in vivo*, indicating that gross systems level perturbation in the underlying networks in culture may contribute to the low concordance.

## Author Summary

Gene expression studies in model systems are widely used for understanding the mechanism of drugs and other perturbations in biological systems. Other researchers have examined the reproducibility of microarray studies between laboratories, or comparing microarrays and/or RNA sequencing. However, no large scale studies have compared results from protocols which differ in minor details, or results generated *in vivo* vs. *in vitro* culture systems thought to serve as useful models. The rat liver is by far the most extensively studied model evaluating effects of drugs and other perturbations, and existing data allowed us to assess the level of concordance between rat liver and rat primary hepatocytes cultured in collagen-coated plates (i.e. “flat” culture) for hundreds of drugs. We found that the mouse liver serves as a better model of the rat liver than do rat primary hepatocytes, even after allowing for differences due to pharmacokinetics. The low concordance observed between rat liver and rat hepatocytes suggests that validating the utility of ‘omics data generated on emerging cell culture approaches (e.g. “organ-on-a-chip”, 3D-printed tissues) using rat cells and comparison to the rat liver may be necessary in order to gain confidence these approaches substantially improve on traditional culture models of human cells.

## Introduction

Transcriptional changes in model systems are often used to elucidate mechanism-based effects of drug treatment and the relevance for humans [1, 2]. While the use of model systems is viewed with skepticism by some, it is common practice to use nonclinical species to inform our understanding of human response (e.g., mouse knockout to human), to extrapolate effects in cell lines to more complex tissues, (e.g., transformed cell lines to tumors), or to use observations in cultured primary cells to understand the behavior of cells in situ in the organ of interest for a disease process (e.g., primary hepatocytes to liver). In each case it is assumed that effects in a less complex system are relevant to a more complex system. Further complicating the use of gene expression to solve these multi-scale and multi-dimensional problems are technical challenges imposed by variability across measurement platforms [3, 4] and differences in experimental protocols (e.g. dose and time) which hamper the ability to aggregate data from multiple sources thereby reducing applicability of existing data. Methods that measure the effects of perturbations across multiple genes, such as gene set analysis (GSA) [5] and co-expression networks [6] may reduce technical and experimental noise while boosting relevant biological signals across data sources [7].

Gene expression profiling has been applied in many areas. Concerns over drug safety, and in particular drug-induced liver injury, have resulted in large projects to establish reference gene expression databases in nonclinical species [8, 9]. Prediction of liver toxicity in humans from nonclinical species is of particular interest and the rat is a commonly used nonclinical species for testing safety prior to clinical development. Thus, measured by number and diversity of drugs, the rat liver is by far the most extensively studied *in vivo* model using gene expression profiling. Calls to eliminate animal tests in favor of human *in vitro* models increase the need to understand the relevance of conclusions from gene expression studies across models and species [10–12]. Several small scale studies have compared *in vitro* vs. *in vivo* gene expression profiles following drug treatment [13–17]. Discrepancies observed in those studies have been attributed to pharmacokinetics [18, 19] and differences in the baseline state of liver vs. its constituent cells in culture [20–22]. However, there are no large scale studies that compare the concordance of *in vitro* and *in vivo* response of hepatocytes to drug exposure.

Previous studies have examined the concordance of gene-expression profiles generated using the same samples analyzed with different microarrays and/or RNA-seq [23, 24]. Since the regulation of gene expression varies across tissues and organisms (“models”) [25], it is also of interest to compare concordance across models. In this work, we take a systems level approach to examine the concordance of transcriptional effects for similar treatments taken from different sources for the same model (i.e. different laboratories), across species (rat, mouse, human) and across cell models comparing *in vitro* to *in vivo*. Leveraging the wealth of time-resolved data within the TG-GATEs database [9], the effect of drug treatment on rat liver is compared to effects observed in mouse liver, cultured rat primary hepatocytes (RPH), cultured human primary hepatocytes (HPH), and HepG2 cells. We compare results using gene expression profiles for individual genes, gene sets or co-expression networks (modules). Modules have been widely used for disease characterization studies [26–29] and reduce the dimensionality of gene expression data while avoiding biases inherent in relying on canonical pathways that dominate gene set enrichment analysis [5]. We examine the absolute level of concordance achieved for each method to understand the impact on mechanistic interpretation (“the most highly induced genes/pathways/modules in RPH are A, B, C and the same genes/pathways/modules are also highly induced in rat liver”), and relative concordance (“are the profiles for a drug in RPH and liver more similar than profiles involving different drugs?”). We refer to the latter as success in “self-identification”; in the absence of high absolute concordance one may still successfully infer properties of a drug (classification) via those of drugs having the most similar expression profiles (i.e. ConnectivityMap-type applications [30–33]). We explore possible causes for the low concordance of *in vivo* vs. *in vitro* profiles for all methods by comparing the baseline state of cultured cells vs. rat and human liver as an estimate of system perturbation. By analyzing whole liver vs. freshly isolated and de-differentiating RPH after various times in culture, we dissect the contributions of non-parenchymal cells resident in liver tissue vs. changes associated with de-differentiation in culture as factors explaining divergence from rat liver gene expression.

## Results

### Repertoire of gene expression profiles in rodent liver and cultured hepatocytes

An extensive characterization of transcriptional effects of drugs and other compounds in rat liver and related model systems have been compiled in two publicly-available resource: the Drug Matrix (DM) [8, 34] and the open TG-GATEs (TG) databases [9]. We analyzed Affymetrix microarray data for 3528 experiments in male rat liver from TG, and 654 similar experiments from DM. An experiment denotes expression results for a unique combination of species, strain, tissue, drug, dose and time, usually obtained by comparing 3 biological replicates in the treatment group to 3 or more replicates in the control group. Treatment groups are always time-matched to controls. To establish concordance with rat liver results across different models, we analyzed 1260 experiments performed in RPH and 941 experiments in HPH from TG, and 268 RPH experiments from DM. We also retrieved 49 mouse liver and 177 HepG2 experiments from the Gene Expression Omnibus (GEO) repository [35], most of which overlap with drugs available in TG and DM. The experiments considered for this work are reported in S1 Dataset.

### Quantitative assessment of drug-induced effects on the transcriptome

Many approaches have been employed for describing drug-induced transcriptional changes at various levels of granularity, including gene-level, gene set analysis (GSA) and co-expression network analysis. Transcript abundance is a well-established factor governing the concordance of microarray and RNA-seq analyses [23]. To avoid including poorly-performing low abundance transcripts, we identified 9071 genes, henceforth described as “liver-expressed genes” having above median intensity in ≥10% of DM rat liver treatment or control arrays. Fold-change values were calculated for each gene by comparing treatment and control samples. In this work, the ensemble of 9071 fold change values is denoted as gene-level analysis. In addition, we performed GSA (using the PAGE algorithm [36]) to identify gene sets enriched among perturbed genes for each experiment, using a subset of the MSigDB collection [5]. Finally, we calculated the eigengene score (or module score) for each of 415 co-expression networks obtained with the WGCNA package [37] applied to the DM rat liver dataset. The eigengene summarizes induction / repression of the underlying module genes, with gene weights proportional to their degree of co-expression. Individual genes, gene sets and modules are described as “features” below.

**Fig 1:**
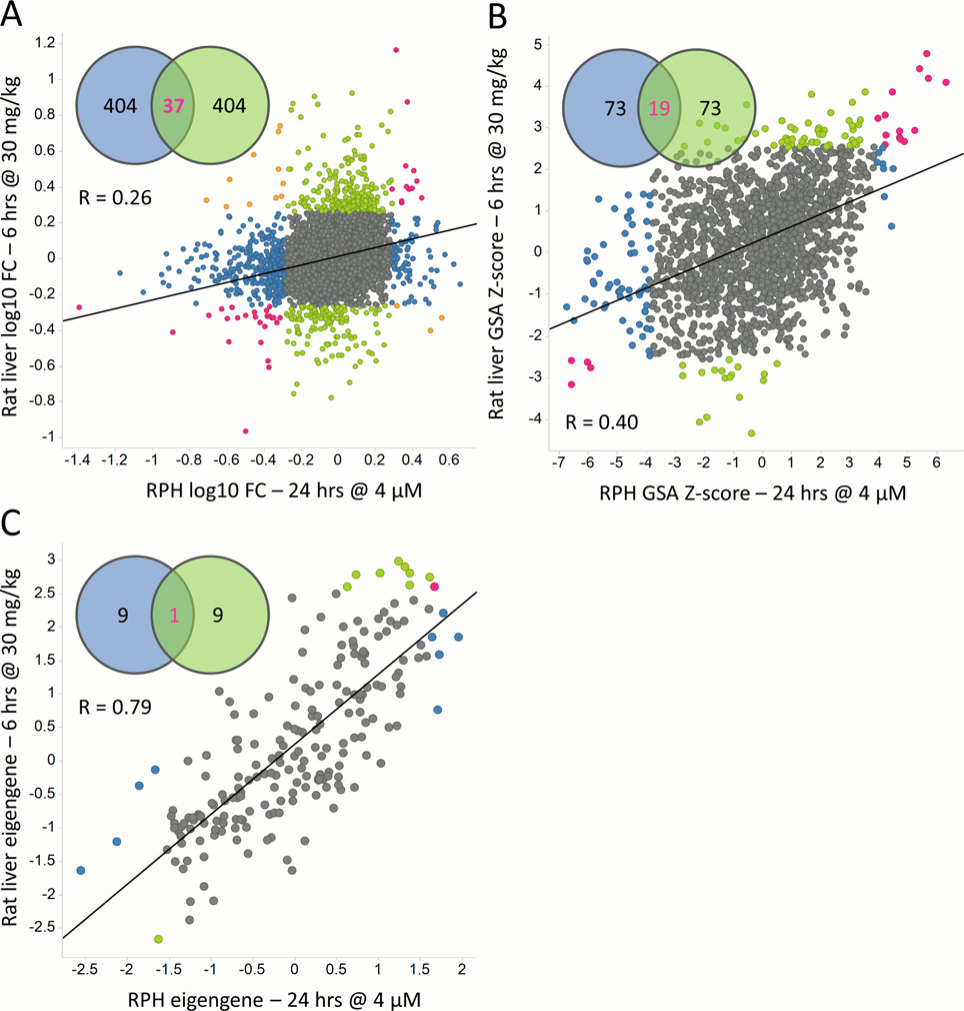
Concordance of azathioprine in rat primary hepatocytes and rat liver. Azathioprine incubated at 4 μM for 24 hours in RPH (X-axis) is compared to rat liver 6 hours after administering a 30 mg/kg dose (Y-axis) using A) 9071 liver-expressed genes, B) GSA analysis of 1840 gene sets and C) 415 co-expression modules. The concordance is quantified using the Pearson correlation coefficient R and percent overlap among the highest 5% of differentially expressed features. Features in blue are in the top 5% for RPH only, those in green in the top 5% for rat liver only, those in pink in the top 5% for both models, and those in orange (only observed here for gene-level analysis) are in the top 5% for both models but induced in one model and repressed in the other. The Venn diagrams denote overlap among the top 5% of features.

To evaluate the concordance of transcriptional changes caused by drug treatment, assessed via gene-level, gene-set and module analysis, we first identified all pairs of experiments that involve the same drug. Each experiment in the pair may involve a different dose, treatment time, system or source of data. Two metrics commonly used in comparing gene expression results were used to assess concordance: the Pearson correlation coefficient and percent overlap among the top 5% of differentially-expressed features (genes, gene sets or modules). Percent overlap was employed for this purpose in the MicroArray Quality Control projects [23, 24]. Fig 1 illustrates the comparison of three methods and two metrics on azathioprine treatment in rat liver and RPH. Comparison of the metrics in the TG data shows that Pearson R and the 5% overlap metric are only approximately correlated. The percent overlap at other thresholds (top 2.5% and top 10%) yield similar results to the 5% threshold (S1 Fig), henceforth, the overlap metric for all analyses uses the top 5% of features.

### Identification of experimental factors accounting for expression profile variation

Within the *in vivo* or *in vitro* setting, there are few drugs studied at the same dose and time in multiple models or sources (13 for TG vs. DM rat liver, 2 for TG vs DM RPH). This severely limits our ability to study concordance across models and sources for identical treatments. We sought to identify factors with small effects on concordance within one system and source, in order to relax our criteria for identifying comparable experiments across models and sources. To this end, we tabulated the concordance of 78,226 pairs of rat liver experiments and 10,008 pairs of RPH experiments from TG involving the same drug. The importance of dose and time difference, study design (repeat vs. single-dose) and overall transcriptional activity caused by treatment were studied using stepwise linear regression (i.e. variables which explain more variation in concordance are those with greater effect on gene expression changes resulting from treatment). Across rat liver and RPH, for any of the three analysis methods or two concordance metrics, differences in time and overall transcriptional activity of the least perturbing treatment explain the largest percentage of variation in concordance (Table 1). Dose differences between treatments explain less than 0.1% of variation in all cases. This suggests that rigorous comparisons across sources (within the *in vivo* or *in vitro* setting) should minimize differences in time, but allow mismatch on dose in order to increase the number of comparable experiments. Further, the level of concordance was strongly influenced by the level of transcriptional activity for the least perturbing of two treatments, as quantified by the average absolute eigengene score across the co-expression modules. Thus, concordance for subtle treatment-induced transcriptional effects is lower than for treatments causing large effects. Our selected measure of transcriptional activity, the average absolute eigengene score (avg. abs. EG), is highly correlated with percent of genes differentially expressed (S2 Fig). Similar conclusions are reached regarding the impact of dose and time differences by tabulation of concordance for adjacent doses or time points in the TG-GATEs study design (S1 Table).

**Table 1:** Percentage of concordance variation explained by treatment characteristics when comparing two different treatments of same drug within TG-GATEs rat liver or RPH.

	Percent overlap top 5% of DE^c^ features			Pearson R		
Parameter	genes	GSA^d^	modules	genes	GSA	modules
	**Rat liver**					
log10 dose ratio	0.09	0.07	0.01	0.10	0.04	0.02
log10 time ratio	24	8	14	24	8.5	14
dosing schedule diff.	1.7	0.8	0.5	1.0	0.42	0.60
smallest avg abs EG^a^	15	15	12	19	11	19
largest avg abs EG^b^	0.7	0.08	0.8	0.06	0.02	0.02
	**Rat primary hepatocytes**					
log10 dose ratio	0.01	0.04	0.00	0.05	0.09	0.01
log10 time ratio	54	26	27	57	28	37
smallest avg abs EG^a^	1.8	3	4.5	6.9	3.1	4.8
largest avg abs EG^b^	5.3	1.8	1.9	0.01	0.03	0.02

Stepwise linear regression was used to build 6 models (2 concordance metrics × 3 feature types) for 78,226 pairs of rat liver experiments and 10,008 pairs of RPH experiments involving the same drug. In each model, the dependent variable is the Pearson R or overlap metric and the independent variables are the parameters shown above. The percent of additional variation explained upon addition of each parameter is shown. Dose and time differences were log-transformed to increase normality of distribution. Differences in dosing schedule was coded as a 3-level categorical variable, indicating whether the paired experiments are both single dose, both repeat dose, or single vs. repeat dose. The overall level of transcriptional changes is modelled as the average absolute eigengene (avg. abs. EG) for ^a^ the least perturbing or

^b^ most perturbing of the two treatments in the pair.

^c^ DE = differentially-expressed

^d^ GSA = gene set analysis

### Concordance in rat and mouse liver

Having identified dose differences as being of lesser importance in determining concordance within the TG rat liver system, we identified comparable experiments in order to assess concordance across sources and systems. The TG rat liver model serves as reference system for comparing drug-induced transcriptional responses in other model systems. We define three levels of stringency in identifying comparable treatments in liver: 1) no time difference and ≤ 5-fold dose difference; 2) ≤ 2-fold time difference and ≤ 10-fold dose difference; 3) no constraint on time or dose differences. Within each comparison category, we identify the most concordant TG rat liver experiment satisfying the constraints. This selection is repeated for both concordance metrics and 3 analysis methods (genes / GSA / modules). Level 2 is arguably a reasonable choice for assessing cross-source concordance, as pharmacokinetic parameters vary across individuals and species. Level 3 analysis offers a very optimistic view of concordance, whereby each experiment is compared to all TG rat liver experiments for the same drug and the most concordant pair retained. Since all drugs were tested at 3 doses and 3, 6, 9 and 24 hours, and most drugs were also tested at 4, 8, 15 and 29 days, this view takes the highest level of concordance across 12 or 24 *in vivo* “snapshots” from TG liver for a given drug. By necessity, we allowed ≤ 1.15-fold time differences (7 vs. 8 day study comparisons, required for GSE43977) for level 1 comparisons from rat to mouse liver, because few experiment pairs across models used exactly the same time point.

Within the constraints defined above, we performed three comparisons using liver transcript profiles: 1) TG rat liver vs. itself, 2) TG rat liver vs. DM rat liver and 3) TG rat liver vs. GEO mouse liver. The first comparison serves as a positive control. This establishes the upper boundary of concordance that can be expected within a large dataset built using a uniform protocol but with information collected over an extended period of time or using different staff and facilities, factors know to influence concordance across microarray experiments even when the same samples are used [3, 4]. The second comparison serves as a robust assessment of cross-source concordance across 33 shared drugs having experiments in both DM and TG liver that match at the highest level of stringency. This represents a “real-world” assessment of concordance, as the DM and TG efforts used the same rat strain (Sprague Dawley) but sourced from different colonies (Charles River Japan vs. Wilmington, MA) with slightly different ages at study onset (6 weeks vs. 7–9 weeks), housed/handled in different facilities and non-identical microarray study protocols [8, 9, 38]. The third comparison begins to address the extent to which gene expression changes observed in one species are relevant to another, by examining transcriptional effects of 10 drugs for which we could find mouse liver experiments matching at the high stringency level in GEO.

In addition to analysis of transcriptional effects via genes, GSA and modules, we considered GSA applied exclusively to REACTOME pathways, and co-expression network analysis using a subset of 216 modules with preservation Z-summary ≥ 5 in TG rat liver (methods). The latter represent the ~50% of modules derived in DM rat liver with the highest degree of validation in an independent dataset. As above, concordance between pairs of experiments is evaluated using the Pearson R and overlap metric. In addition, we report the probability that a given level of concordance between experiments involving the same drug exceeds the level seen for random pairs involving different drugs (methods). This is especially relevant for CMap-type analyses, where the goal is not mechanistic understanding of drug effect, but the inference of drug properties via those of well characterized drugs with similar gene expression profiles [30–33]. For simplicity below, we refer to this classification metric as successful “self-identification” vs. random pairs involving different drugs: the gene expression profile for a given drug is more similar to other profiles involving the same drug (in another system, or at a different dose or time) than profiles involving different drugs.

**Fig 2:**
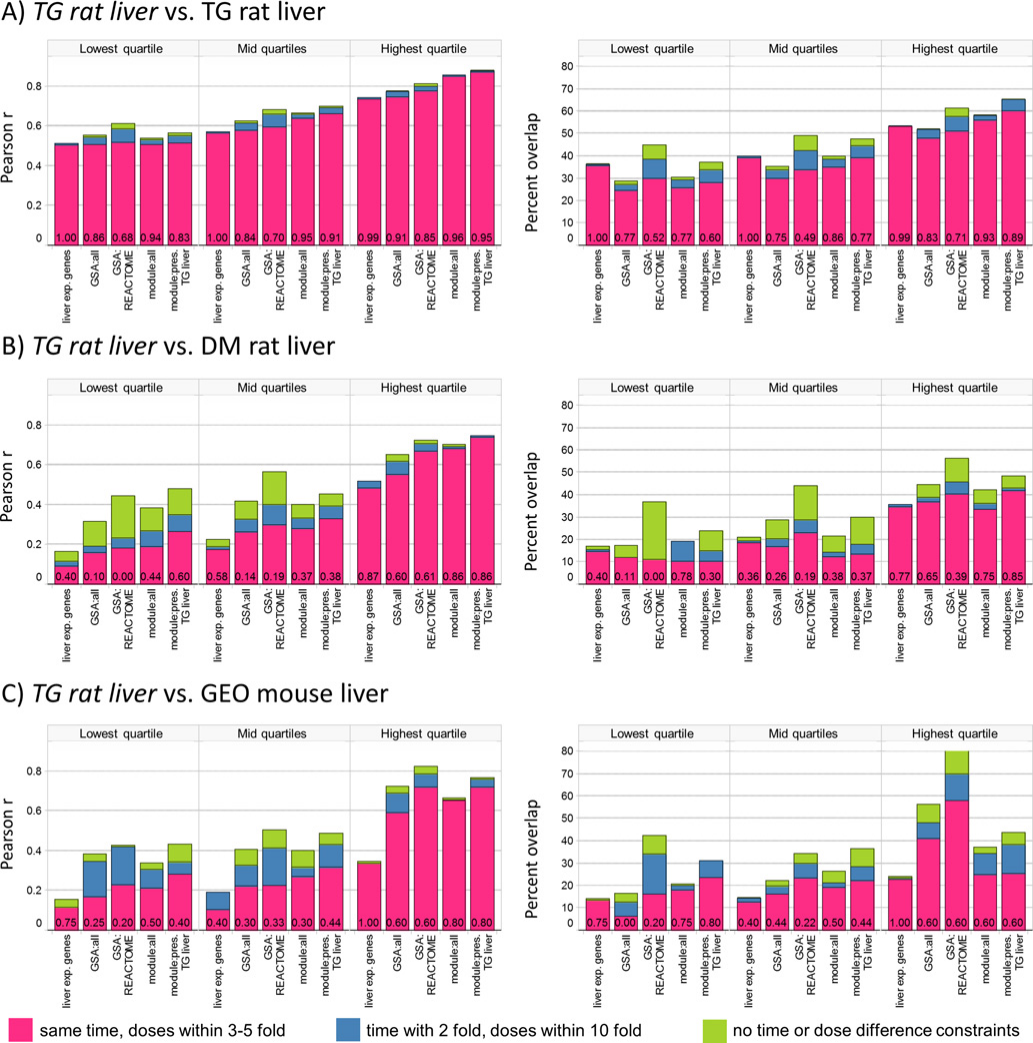
Concordance of drug-induced effects in rat and mouse liver. Pairs of experiments involving the same drug are compared via the Pearson R and percent overlap metrics, and average concordance is calculated between TG rat liver (reference set) and A) TG rat liver (within-source), B) DM rat liver (cross-source) and C) GEO mouse liver (cross-source and cross-species). Concordance is shown additively for 3 levels of constraint on dose and time differences: experiments at the same time point and doses within 5 fold (pink), time within 2 fold and doses within 10 fold (blue), no constraint on doses or time (green). In each case, experiments are compared to TG rat liver and the most concordant experiment selected within the level of constraint. Only experiments that have a match to TG rat liver at the most constrained level are considered at the other levels. Because concordance can only improve upon removing dose and time constraints, results are shown additively with the total bar height denoting the concordance achieved with no constraints. Numeric values represent the probability that the given level of concordance exceeds that of random pairs involving different drugs for the most stringent level considered (pink bars). Experiment pairs are separated into 3 ranges based on the level of transcriptional activity for the least perturbing of two treatments (lowest quartile have avg. abs. EG ≤ 0.28, highest quartile have avg. abs. EG > 0.46; thresholds selected using 3528 TG rat liver experiments).

Concordance of experiment pairs is tabulated across quartiles of transcriptional activity for the least-perturbing of two treatments in the pair, given the importance of this variable in explaining intra-source concordance (Fig 2, S2 Dataset). This can be seen from comparisons within TG rat liver, where all methods yield higher concordance with increasing levels of transcriptional activity (Fig 2A). Improvement in concordance when relaxing constraints on dose and time differences (pink -> blue -> green bars) is minimal within-source, indicating that the most similar experiments by gene expression tend to be the most similar experiments by dose/time. All methods have high self-identification probability.

The TG vs. DM liver comparison reveals lower concordance than the within-source trends described above, coupled to greater improvement in concordance when relaxing constraints on dose and time differences between experiments (Fig 2B). The dependence on level of transcriptional effects is larger, with *ca*. 50% or less success at self-identification in the lower quartiles. The comparison between TG rat and GEO mouse liver is broadly similar for GSA and modules, especially when allowing less stringent matches on dose and time (Fig 2C; pink+blue bars). Gene-level concordance is lower relative to GSA or modules for the cross-species comparison on both metrics (within each of 18 combination of two metrics, 3 ranges of avg. abs. EG and 3 dose/time stringency levels, p < 0.05 for 9 GSA:all vs. gene comparisons and 12 module:all vs. gene comparisons using one-sided t-tests with unequal variance). In contrast, the high performance of gene-level analysis for self-identification between rat and mouse liver indicates that appropriate inferences are made by CMap analyses (i.e. low absolute concordance, but high enough to associate the same or similar drugs across models).

The importance of considering both the quantitative level of concordance, along with probability of self-identification is illustrated in S3 Fig. The threshold of similarity required to exceed that observed for random experiment pairs differs for each combination of method and metric. This threshold also increases as a function of overall transcriptional activity, since the concordance for random pairs involving different drugs increases as a function of avg. abs. EG (S2 Table). For experiments in rat liver in the mid-range of transcriptional activity, the level of similarity on the Pearson metric required to achieve a threshold of 50% success at self-identification increases from approximately 0.2 for gene-level analysis to 0.55 for GSA:REACTOME. As such, the apparent advantage of GSA:REACTOME on the percent overlap metric (Fig 2) is not sustained when considering success at self-identification (S2 Dataset).

### Concordance between rodent liver and cultured hepatocytes

A significant challenge in comparing results from gene expression profiling between *in vivo* and *in vitro* experiments arises from the confounding effects of *in vivo* pharmacokinetics. This is elegantly described for methapyrilene-treated rat liver and rat hepatocytes by Schug et al., where concentration decreases slowly in culture but rapidly (and at different rates) in blood from arterial, liver and portal veins [19]. A further dilemma in aligning *in vivo* vs. *in vitro* exposures concerns differences in non-specific binding between plasma and media [39], neither of which have been measured in the TG-GATEs initiative. We adopted the use of an optimistic comparison, whereby each *in vitro* experiment is compared to all TG rat liver experiments for the same compound and the most concordant pair retained. We assume that one or more of these 12 or 24 *in vivo* “snapshots” should reasonably reflect the drug / time conditions under study *in vitro*.

**Fig 3:**
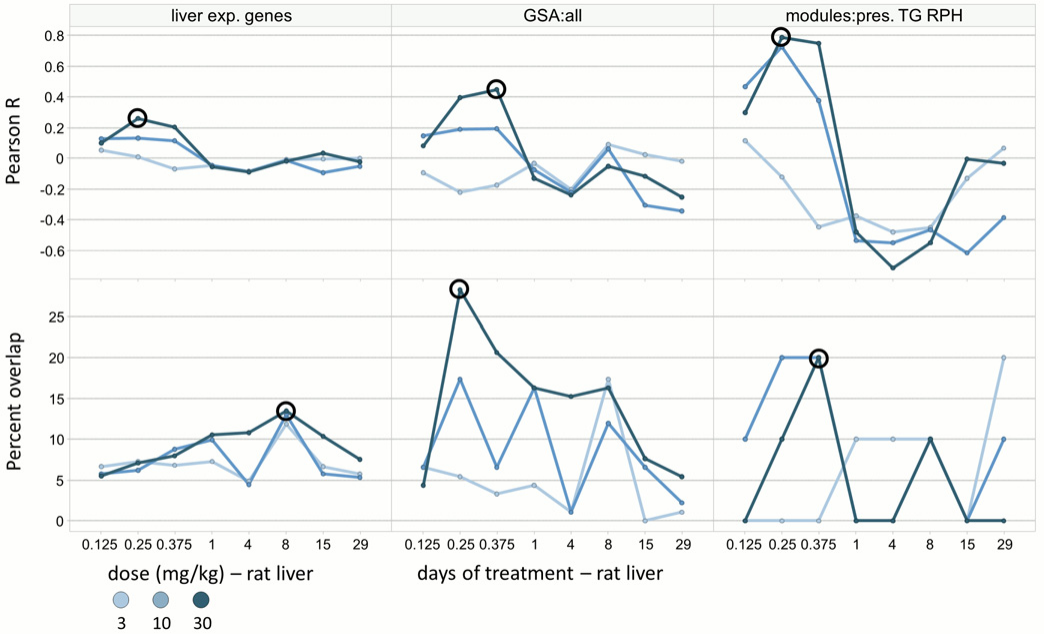
Comparison of *in vitro* vs. *in vivo* treatment effects for azathioprine. Transcriptional effects after treatment of rat primary hepatocytes with 4 μM azathioprine for 24 hours were compared to 24 different *in vivo* azathioprine experiments in rat liver. The low, medium and high *in vivo* doses are denoted with separate lines, and concordance is assessed vs. time using 6 analysis methods (two concordance metrics and genes / GSA / module analysis). The circled point denotes the most concordant *in vivo* dose / time condition for each of the 6 analysis methods.

The approach is illustrated for 4 μM azathioprine treatment in RPH 24 hours after drug administration. When using the Pearson metric, the most similar *in vivo* profile is the high dose at 6 hours for gene and module analysis, vs. the 9 hour time point when using GSA (Fig 3). Using the overlap metric selects a similar *in vivo* condition for GSA and modules, but the 8 day condition for gene-level analysis. We selected azathioprine as a case where the correlation (using the Pearson metric) oscillates from positive to negative to minimal, a behavior that reflects the dynamic nature of biological response patterns *in vivo* for acute *vs*. chronic dosing. This example serves to illustrate a key difference between the metrics: Pearson correlation can be negative, conveying a reversal of states (which has been successfully used for drug repurposing[31]), while percent overlap is limited to a range between 0 and 100 and does not distinguish a situation where no genes overlap between two experiments from one where they overlap strongly but change in different directions.

**Fig 4:**
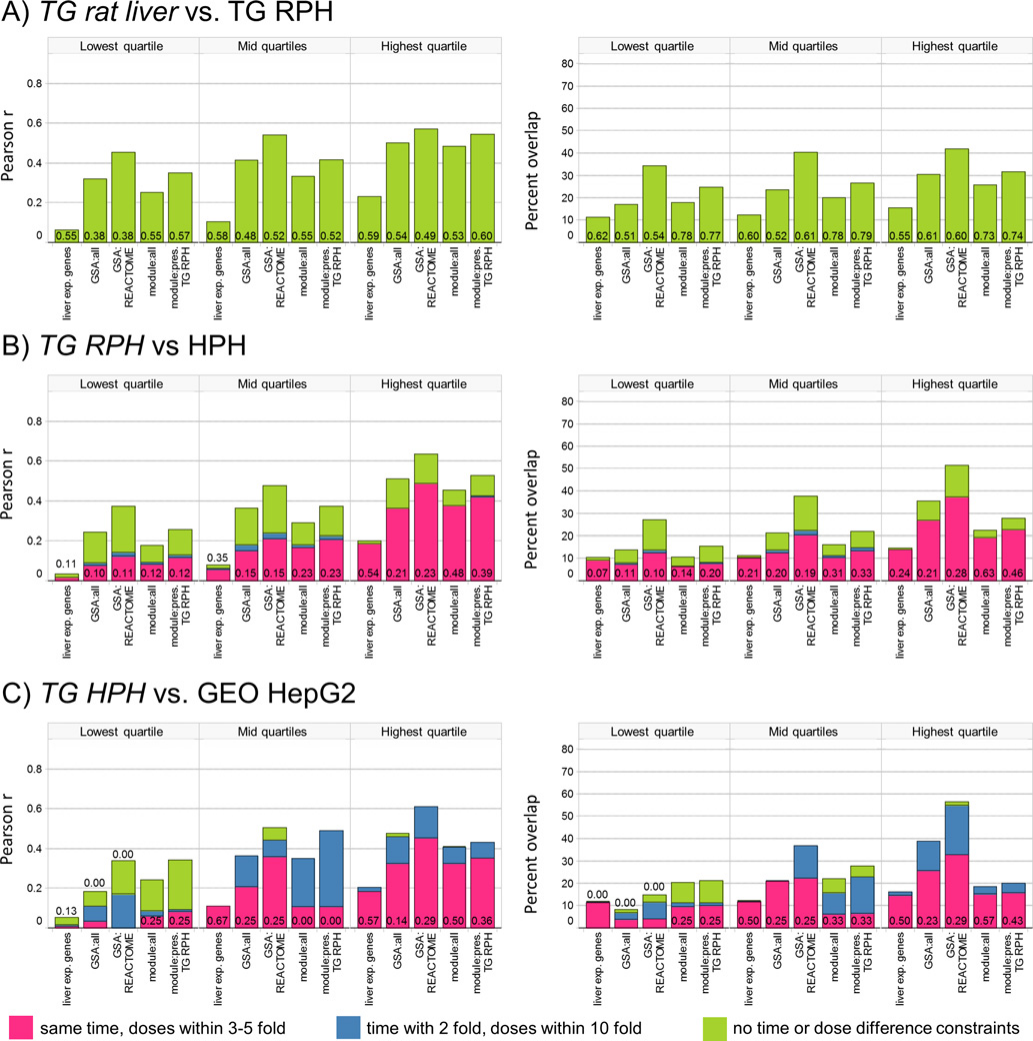
Concordance of drug-induced effects in cultured hepatocytes. Pairs of experiments involving the same drug are compared via the Pearson R and percent overlap metrics, and average concordance is calculated between A) *TG rat liver* vs. TG RPH, B) *TG RPH* vs. TG HPH and C) *TG HPH* vs. GEO HepG2. The data source shown in italics is the reference for each comparison, to which experiments from the other source are compared. For A), only the optimistic comparison is performed because dose / time are not comparable *in vivo* vs *in vitro*. Refer to Fig 2 caption for details.

We used the TG rat liver and RPH experiments to assess whether drug response in cultured rat hepatocytes and rat liver are similar. This comparison is of high interest since the drug lots and experimental procedures for microarray analysis are the same in both experimental models, minimizing the role of technical differences in our assessment. For each of 1255 TG RPH experiments involving 140 unique drugs, we determined the most similar TG rat liver experiment and tabulated the average concordance across methods (Fig 4A). We analyze separately a subset of 207 modules with preservation score ≥ 3 in TG RPH, representing ~50% of modules with higher confidence of co-regulation in culture. Concordance measured on both metrics is lower than the equivalent within-source comparisons for TG rat liver (Fig 2A) and TG RPH (S4A Fig). As observed for rat vs. mouse liver, GSA and modules outperform gene-level analysis; differences for both metrics in each of the 3 ranges of avg. abs. EG are all statistically significant at p < 1e-11 when comparing GSA and modules to genes (one-sided t-tests with unequal variance). Low *in vivo* vs. *in vitro* concordance is also seen when comparing 114 DM RPH experiments to TG liver (S4C Fig). We investigated whether concordance varied across pharmacological classes represented with 3 or more drugs in TG using modules preserved in RPH, the top performing method when considering absolute concordance and probability of success at self-identification (S3 Table). Although we find little evidence of concordance variation across classes, this observation should be tempered by the very limited redundancy of drug classes in TG (and the diversity within classes, e.g. grouping together all non-steroidal anti-inflammatory drugs). The highest *in vivo* vs. *in vitro* similarity is achieved for cycloheximide, galactosamine, tunicamycin, ethionine, azathioprine and 1-napthtyl isocyanate, a group of structurally and pharmacologically diverse agents.

Lower concordance between rat liver and RPH could arise from a combination of pharmacokinetics or differences in the baseline states of hepatocytes affecting how they respond to perturbation. Comparisons involving different *in vitro* models mostly eliminate the impact of pharmacokinetics, as drug exposures from common *in vitro* experiments do not change substantially over 24 hours (compound dissolved in culture media serves as a buffer) [19]. We compared drug-induced expression changes between TG RPH and TG human primary hepatocytes (HPH), comparing each HPH experiment against 12 TG RPH experiments (3 doses × 4 time points). As done for the liver comparisons, concordance is assessed at 3 levels of restriction on dose and time differences, and the most favorable comparison retained at each level (Fig 4B). A similar analysis was performed by comparing 25 HepG2 experiments from GEO involving 18 drugs to 12 TG HPH experiments for each drug (Fig 4C). Concordance for all methods is low, whether considering absolute concordance or success at self-identification. This result is unlikely to stem from differences in cytotoxicity at a given concentration, since most drugs tested in our hands exhibit similar effects on cell viability in RPH and HepG2 cells (S4 Table). A very limited comparison between rat and mouse primary hepatocytes for 5 drugs, used at similar doses for the same treatment duration, yields qualitatively similar conclusions: the most concordant profiles are those from treatments associated with higher levels of transcriptional activity, yet concordance is generally low across these in vitro models (S5 Table).

### Comparison of baseline expression across systems

**Fig 5:**
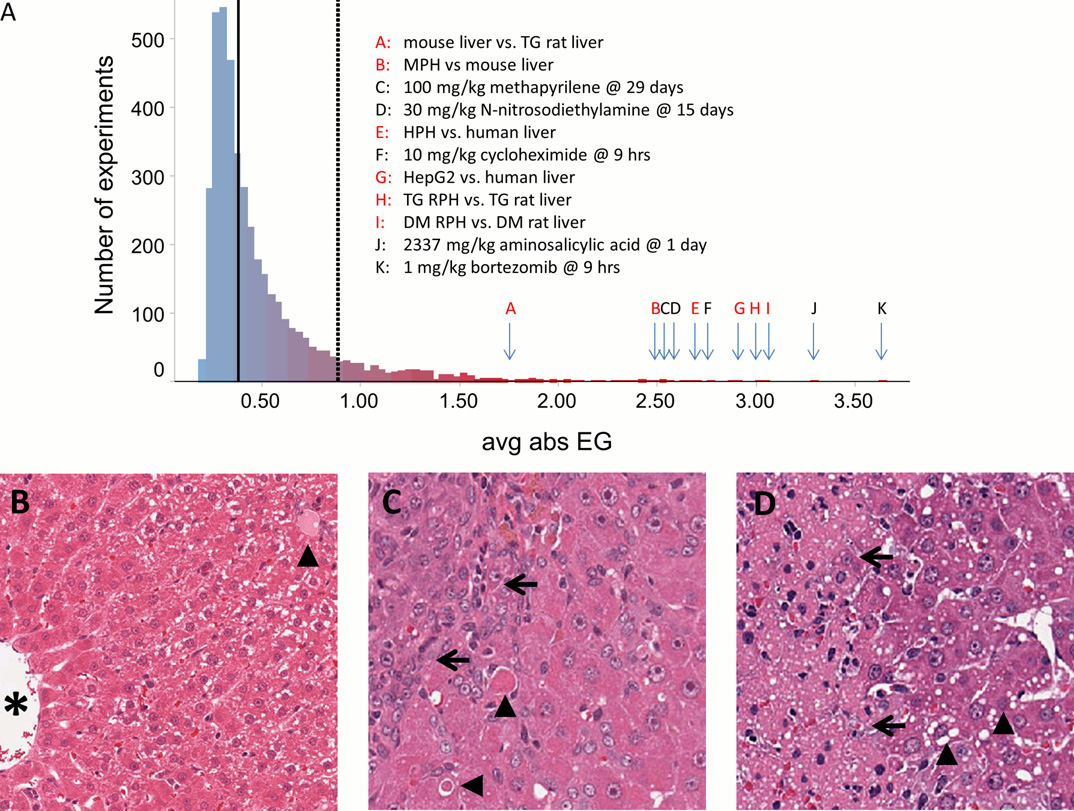
Transcriptional effects of cell culture in the context of rat liver perturbations achieved with drug treatment. A) Distribution of average absolute eigengene scores for 4182 rat liver drug treatments from DM and TG, and 6 baseline expression comparisons of control samples (shown in red text in the inset). The solid and dashed vertical lines denote the median and 90^th^ percentile values for avg. abs. EG. Rat liver histology sections: B) Unaffected liver. A central vein (asterisk) and small portal triad (arrowhead) are marked. Mild hepatocellular glycogen (recognized by cytoplasmic pallor) is apparent in the periportal region. C) Liver histology 1 day after the last dose of methapyrilene administered at 100 mg/kg for 29 days. Biliary hyperplasia (to the left of the arrows), correlating with increased alkaline phosphatase (ALP) and γ-glutamyltransferase (GGT) activities (S7 Table), and hepatocellular apoptosis/single cell necrosis (arrowheads) are noted. Hepatocytes in the right side of the image are hypertrophied and exhibit prominent anisonucleosis. D) Liver histology 1 day after bortezomib administration at 1 mg/kg. Acute hepatocellular necrosis (left of the arrows) and mild hepatocellular vacuolation (arrowheads) are noted. Images were extracted from the Open TG-GATES website (http://toxico.nibiohn.go.jp/english/).

Given that within-source comparison performed well for all analysis methods, we investigated other sources of variation across experimental models by comparing their baseline states, i.e. basal level of gene expression before perturbation with drug treatment. We find that baseline gene expression in control samples is highly correlated across sources for the same system (TG vs. DM rat liver, TG vs. DM RPH; Tables 2 and S6). We also find higher correlation between expression in liver and primary hepatocytes of the same organism in culture versus liver-to-culture expression comparisons across organisms: i.e., rat liver expression is more correlated with RPH expression, than with mouse or human liver; likewise for human liver vs. HPH and mouse liver vs. MPH. However, viewing differences in relative terms (i.e., treating liver as ‘control’ and culture as the ‘perturbation’) reveals that the transcriptional impact on hepatocytes in going from liver to culture is comparable in magnitude to the effect of administering highly toxic treatments to rat liver causing marked changes in liver morphology (Fig 5). It is noteworthy that the comparison of GEO mouse liver to TG rat liver appears less dramatic on this basis (avg. abs. EG = 1.75) compared to the baseline expression correlation. Thus even in cases where simple gene level correlation suggest that two models are similar, the underlying degree of perturbation reflected by changes in co-regulation behavior of genes, and by analogy the biology associated with those genes, are highly perturbed.

**Table 2:** Pairwise correlation of baseline gene expression in untreated liver or hepatocytes.

	DM rat liver	TG rat liver	GEO mouse liver	GEO human liver	DM RPH	TG RPH	TG HPH	GEO MPH	GEO HepG2
DM rat liver	-	0.99	0.83	0.75	0.90	0.91	0.70	0.77	0.63
TG rat liver	0.99	-	0.82	0.75	0.90	0.91	0.70	0.76	0.62
GEO mouse liver	0.83	0.82	-	0.77	0.78	0.77	0.73	0.91	0.66
GEO human liver	0.75	0.75	0.77	-	0.71	0.71	0.87	0.72	0.77
DM RPH	0.90	0.90	0.78	0.71	-	0.98	0.78	0.81	0.70
TG RPH	0.91	0.91	0.77	0.71	0.98	-	0.77	0.80	0.69
TG HPH	0.70	0.70	0.73	0.87	0.78	0.77	-	0.76	0.88
GEO MPH	0.77	0.76	0.91	0.72	0.81	0.80	0.76	-	0.70
GEO HepG2	0.63	0.62	0.66	0.77	0.70	0.69	0.88	0.70	-

Correlation of gene-level log intensities at baseline (untreated samples) across model systems and sources. The number of genes varies between ca. 13,000 and 16,000 depending on ortholog relations and/or presence of genes on specific Affymetrix microarrays

### System level perturbations and differentiation state of hepatocytes in culture

To understand the causes and nature of the underlying perturbation reflected in baseline perturbations noted for the *in vitro* models, we investigated both the contributions of non-parenchymal cells to *in vivo* transcript profiles and changes associated with the adaptation of the hepatocytes to culture, often characterized as a de-differentiation process [40, 41]. Non-parenchymal cells comprise approximately 6 percent by volume of the liver (40% by cell count) [42] and cell population differences between liver and enriched freshly isolated hepatocytes may account for some level of discrepancy between the transcriptional response in whole liver vs. response in cultured hepatocytes.

**Fig 6:**
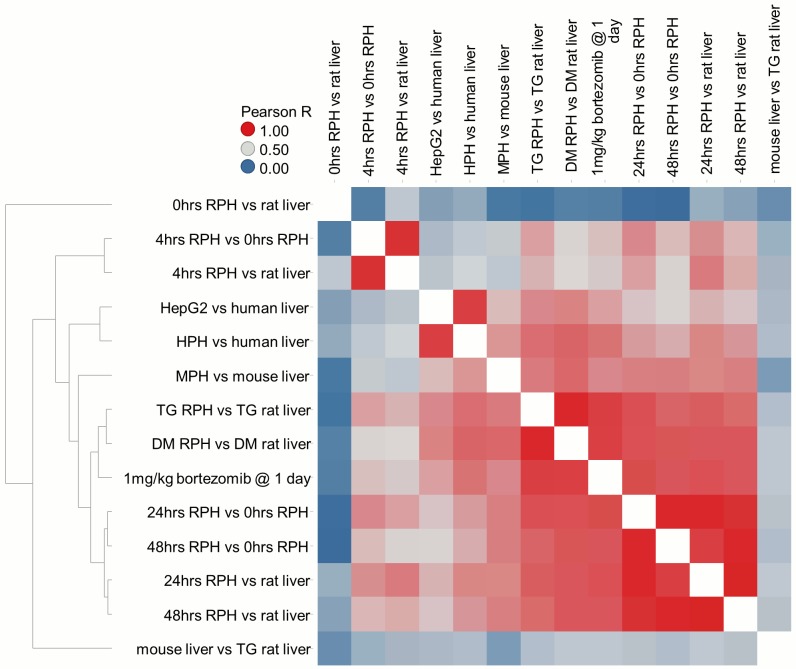
Correlation analysis of transcriptional changes in hepatocyte cell culture. Co-expression network analysis of expression changes when comparing mRNA between cultured hepatocytes vs. liver or immediately after hepatocyte isolation (vs. 0hrs RPH). Cells are colored on the Pearson correlation coefficient R obtained by comparing module scores for two conditions. Conditions are clustered to group those showing similar scores across 415 modules, revealing broad similarity following 24–48 hours in culture for RPH, HPH, MPH and HepG2 cells from various sources.

We first prepared enriched rat hepatocytes, using a standard liver perfusion protocol, and determined the transcript profiles immediately after isolation (0 hour time point), and after 4, 24 and 48 hours of culture on collagen-coated plates. We compared the hepatocyte transcript profiles to those from 3 samples of intact liver serving as a control. We performed two normalizations for the 4, 24 and 48 hour samples: 1) using control liver to assess the impact of a reduction in non-parenchymal cells relative to normal liver; and 2) using the 0 hour isolated hepatocyte time point as control to determine change associated with alteration in hepatocellular phenotype in culture. Both module or gene-level analysis demonstrate high concordance between the responses noted in 24 or 48 hour RPH vs. liver in our experiment, and those obtained by comparing vehicle-treated RPH and liver control samples in DM and TG, despite differences of time in culture, culture protocols and the use of vehicle treatment for the DM and TG samples (Fig 6; S3 Dataset). This suggests a high degree of similarity between the underlying biological perturbations in our experiment and those in both DM and TG studies.

The transcriptional impact of removing the bulk of non-parenchymal cells (0 time vs control liver) is relatively large in the context of rat liver treatments (avg. abs. EG 0.64, or 81^th^ percentile among 4182 DM and TG rat liver experiments). However, equivalent comparisons at 4, 24 and 48 hour samples indicate substantially larger perturbation (avg. abs. EG scores of 2.1, 2.4 and 2.5, respectively, ≥99^th^ percentile). Differentially expressed genes at the 0 hour are consistent with attribution to cell type differences since a variety of pathways and GO-biological process terms not generally associated with hepatocytes were enriched in down-regulated genes, e.g. hemoglobin complex, extracellular matrix, immune response, leukocyte migration). Liver is a site for extramedullary hematopoiesis (hemoglobin complex), stellate cells remodel extracellular matrix components in liver, and there are a variety of liver resident immune cells among the non-parenchymal liver cell population [42]. Pathways and terms enriched at the 4, 24 and 48 hour time points indicate dramatic differences in activity of fundamental cellular process reflecting cell activity, movement and cytoskeletal structure often associated with de-differentiation (e.g. up-regulation of RNA processing, ribosome biogenesis, translation, focal adhesion and microtubule cytoskeleton; down-regulation of fatty acid oxidation, CYP450 drug metabolism; S4 Dataset). Changes in expression at 24 or 48 hours in RPH culture are highly correlated, whether they are compared to the 0 hour time point or rat liver, suggesting the cells stabilize after 24 hours in culture. Taken together, this indicates that de-differentiation of hepatocytes in cultures accounts for most of the difference relative to rat liver rather than differences in cell type composition. Further, effects observed in comparing cultured rat hepatocytes to rat liver are broadly similar to those seen for cultured human hepatocytes vs. human liver and mouse hepatocytes vs. mouse liver (Fig 6; S3 Dataset). This suggests that the processes responsible for hepatocyte de-differentiation in culture are similar across organisms.

To determine if the changes seen upon de-differentiation in culture are relevant to any biological states in rat liver, we compared the changes noted in culture to various liver states after drug treatment in TG and DM. Of note, the transcriptional effect of cell culture on hepatocytes is highly correlated with the effect of 24 hours of bortezomib treatment in rat liver (Pearson r = 0.85; Fig 6) and with the treatments included in Fig 5 (Pearson R of 0.67 for cycloheximide, 0.77 for carbon tetrachloride, 0.66 for methapyrilene, and 0.69 for aminosalycilic acid). These data suggest that the perturbations seen upon adaptation of hepatocytes to culture resemble biological states adopted by hepatocytes as a response to drug-induced liver injury *in vivo*. Thus, treating hepatocytes in this type of simple culture models may be more analogous to dosing animals with an injured liver than they are to responses in a naïve animal.

## Discussion

Deconvoluting from phenotypic information to mechanistic understanding is an essential component of drug discovery and requires extrapolation of results from simple to more complex systems and from one complex species to another. In the former, reducing a complex system to a testable model, such as cells in culture, yields mechanistic results that are extrapolated back to a complex tissue or whole organism. In the latter, information from experiments in one species, e.g. rats or mice, are used to infer behavior of another complex system or population, humans. These comparisons are complicated by the multi-scale nature of these systems as well as differences in the dimensions of dose and time. Inaccurate translation results in failure to predict biological responses in human subjects [43]. Systems approaches are suited to understanding individual changes, such as changes in mRNA levels, yet there are few studies that address concordance of mechanistic information across levels of complexity. We addressed this problem two ways, by evaluating concordance across the evolutionary dimension (mouse, rat and human) and across scales of complexity (cells to tissues) in the same species. We defined components of variation (dose, time, overall transcriptional activity) and evaluated concordance with differing level of stringency using commonly-applied methods (gene-level and GSA) and co-expression network (module) analysis. The latter approach has not been described for toxicogenomics studies, but represents a class of methodologies frequently used in disease characterization studies [26–29].

A major goal of gene expression analysis is to assemble a specific list of genes, pathways or modules that are differentially expressed in response to drug treatment and reflect meaningful biological response. Understanding the degree to which the list, and therefore the mechanistic interpretation, would change as a result of using other measurement technology (microarrays, RNA-seq), sample, species or culture conditions is important. Our findings on concordance build upon comprehensive analyses published with regards to measurement technology [3, 4, 23, 24] and are summarized as follows:

- When comparing results for the same model and source (rat liver or RPH within TGGates; Figs 2A and S4A), high concordance is seen for all methods and both metrics, with Pearson R of ca. 0.8 and percent overlap of ca. 60% or higher achieved in the highest quartile of transcriptional activity. These results are consistent with the MAQC findings on three compounds (S8 Table), where approximately 70% concordance was reported and there was little difference between gene-level and pathway analysis.
- Across all comparisons, the level of concordance depends on the overall level of transcriptional activity caused by drug treatment. It should be noted that this is not a statement about mRNA abundance of the underlying genes [23], but the magnitude by which abundance changes in response to treatment. This observation has important implications, as the concordance reported here and in MAQC for treatments causing significant transcriptional perturbation may be significantly higher than the concordance for applications in pharmacology, where non-toxic doses are used and transcriptional perturbations are smaller.
- When comparing results for the same model, but across sources (Figs 2B and S4B), the level of concordance seen within-source is approached only for treatments in the highest quartile of transcriptional activity. The use of GSA and modules, methods which aggregate results across multiple genes, yield modestly higher concordance than gene-level analysis. Similar results are obtained when comparing rat liver to mouse liver, except for gene-level analysis where notably lower concordance is obtained (Fig 2C). While caution is warranted due to the relatively small size of the mouse liver dataset overlapping with rat liver (22 GEO experiments involving 10 unique drugs), this suggests that the coarse-grained view obtained by reducing the dimensionality may be helpful in increasing confidence that findings in one species apply to others.
- Lower concordance is observed when comparing expression profiles from rat liver and rat primary hepatocytes (RPH), relative to comparison across rat liver datasets or from rat to mouse. Under the optimistic scenario, where each *in vitro* experiment was compared to 12 or 24 *in vivo* experiments, Pearson R of ca. 0.55 and percent overlap of 30–40% are achieved with GSA and modules in the highest range of transcriptional activity, with significantly lower concordance for gene-level analysis (Fig 4A). Similar results are obtained when comparing gene expression profiles from *in vitro* systems (RPH vs. HPH, or HPH vs. HepG2; Fig 4B and 4C) suggesting that inherent differences in the models, rather than pharmacokinetics, contribute to lower concordance. Comparison of concordance from Figs 2, 4 and S4 without constraint on dose or time differences (most optimistic) are shown together in S5 Fig. These results are consistent with those reported by Lee at al., where the application of topic modelling to the TG rat liver and RPH data found no overlap of profiles from *in vitro* vs. *in vivo* treatments among the most similar 1% of experiment pairs [44].
- When a library of reference profiles is available, comparing the profile of a treatment against the library to infer its properties from well-studied drugs is a common approach (e.g. CMap analysis [30–33]). The absolute level of concordance is unimportant, provided that the most similar reference profiles do in fact belong to similar drugs (similar pharmacology, indications, etc.). For this task, gene-level and module analysis have high success rates for self-identification within one system (a drug, perhaps at a different dose or time, is most similar to profiles obtained from the same drug in the reference library; S9 Table). GSA, especially on REACTOME pathways, performs notably worse. This suggests that the biological processes captured by canonical pathways are commonly perturbed across diverse drugs, reducing their utility for identifying drugs with similar properties. Mirroring our findings on overall concordance, success at self-identification when comparing rat liver to RPH, or between cultured cells is ca. 50%, suggesting that care should be applied when leveraging reference profiles across models.

Several studies have analyzed TG data, with the goal of identifying genes or pathways that may be used to predict a phenotype (e.g. carcinogenicity). Zhang et al. identified 4 acute-response genes that predict the occurrence of cell death *in vitro* or liver injury *in vivo* [45]. El-Hachem et al. analyzed the data using GSEA to identify REACTOME pathways which can be modulated across systems and associate with carcinogens or PPAR alpha activators [46]. There is a large body of literature which has successfully demonstrated prediction of *in vivo* properties, especially carcinogenicity, from *in vitro* expression results (e.g. [47–50]). The goal of these studies is to identify a small number of genes or pathways that predict a specific *in vivo* property, with the possibility of constraining the selection to genes that behave consistently across several systems (e.g. different culture models, different species, etc.). Here we examine a different application of gene expression profiling, of relevance to a scenario where one uses gene expression profiling to understand mechanistic effects of a drug not necessarily tied to a specific phenotype. Effects at the level of the whole genome are considered, not just a subset of high performing genes identified in situations where a researcher has the benefit of large numbers of samples available for model training. As such, the findings reported here do not inform on the applicability of predictors derived in one system to other systems.

Our analysis of baseline states (expression level in untreated samples) underscores the dramatic transcriptional changes in culture, comparable in magnitude to very toxic rat liver treatments. Comparison of various *in vitro* models, where the impact of pharmacokinetics is minimal, suggests that the different baseline states of underlying biological systems in these models accounts for differing response upon drug treatment. To our knowledge, there are no published datasets describing drug-induced transcriptional effects in human liver, limiting our ability to assess the utility of advanced culture models for *in vitro* studies on human cells (e.g. “organ-on-a-chip”, 3D-printed tissues, iPS cells, etc). Nonetheless, based on the available rodent data, the scope for improvement over “flat” cell culture (culture of cells adhered to collagen-coated plates) is evident. Three dimensional culture models have shown improvement in the concordance vs. liver of cell viability assessments [51–53] or gene expression changes for nanomaterials [54]. Our results suggest that demonstrating improved concordance across a range of drug treatments in the context of the well-studied rodent liver seems warranted. The correlation of transcriptional changes that occur when rat and human hepatocytes differentiate in culture suggest that improvements in modelling the rodent liver using cultured rodent cells may be transferrable to the study of human liver effects using human cells.

## Materials and Methods

### Ethics statement

The studies on rat primary hepatocyte de-differentiation were conducted in accordance with the Guide for the Care and Use of Laboratory Animals as adopted and promulgated by the U.S. National Institutes of Health, and were approved by the Lilly Animal Care and Use Committee. Animals were given an ip injection of Na pentobarbital (275 mg/kg) and sacrificed via exsanguination. Rodent and de-identified human gene expression data from Drug Matrix, open TG-GATEs and the Gene Expression Omnibus (GEO) repository are freely available to the public. No institutional review board approval was sought to analyze those data.

### Isolation and *in vitro* culture of rat primary hepatocytes

Rat hepatocytes were isolated from three male Sprague Dawley (SD) rats using the method of Berry and Friend [55] and Seglen [56]. During the isolation and prior to perfusion, a lobe of liver was tied off and homogenized in Trizol for RNA isolation according to the manufacturer’s instructions (Invitrogen). This served as the liver in situ reference sample. Cells were isolated and samples from the cell pellet (time zero) and cells cultured for 4, 24 and 48 hours were placed in Trizol for RNA isolation. Cells were cultured in William’s E media supplemented with glutamate, gentamicin, insulin, transferrin, dexamethasone and serum (10% FBS) for 4 hours and then exchanged with serum free media with the same constituents. Three biological replicates were generated for each group (one from each rat). Each biological replicate was analyzed via 3 technical replicates, for a total of 9 array hybridizations per group.

### RNA Processing and Array hybridization

All RNA was cleaned using Qiagen RNeasy columns (Qiagen) and evaluated on an Agilent Bioanalyzer (Agilent). Samples for mRNA profiling studies were hybridized to Affymetrix Rat Gene Expression 230–2 arrays according to the standard Affymetrix protocol. Briefly, total RNA was used for preparation of biotin-labeled cRNA. Labeled cRNA was fragmented and used for array hybridization. Arrays were washed and stained with streptavidin-conjugated phycoerythrin on an Affymetrix FS450 Fluidics station. The arrays were scanned on an Affymetrix GeneChip Scanner. A summary of the image signal data, detection calls, and gene annotations for every gene interrogated on the array was generated using Affymetrix GeneChip Command Console MAS 5.0 algorithm with all arrays scaled to 500. One array from each of the 4 and 48 hour time points did not meet standard microarray QC metrics and were excluded from analysis. RNA was then labeled and hybridized to RG230-2 microarrays (Affymetrix) according to the manufacturer’s instructions.

The data has been deposited in NCBI's Gene Expression Omnibus [35] and are accessible through GEO Series accession number GSE74903 http://www.ncbi.nlm.nih.gov/geo/query/acc.cgi?acc=GSE74903).

### TG-GATEs, Drug Matrix and GEO data retrieval and processing

Affymetrix RG230-2, MG430-2 and HGU133-2 microarray data was obtained from multiple sources. Throughout this work, an “experiment” denotes one species / tissue / drug / dose / time combination, usually analyzed with 3 biological replicates and always compared to a time-matched vehicle or DMSO control. The National Institute for Environmental Health Sciences (NIEHS) hosts the Drug Matrix database [8] (DM) on its website (https://ntp.niehs.nih.gov/drugmatrix/index.html). We retrieved CEL files for 654 male rat liver and 268 rat primary hepatocyte (RPH) experiments on RG230-2 microarrays. The TG-GATEs database (TG) consists of approximately 160 drugs and reference compounds used to generate gene expression profiles in rat liver, rat kidney, rat primary hepatocytes (RPH) and human primary hepatocytes (HPH) [9] (http://toxico.nibiohn.go.jp/english/). We retrieved CEL files for 3528 male rat liver experiments (single and repeat dose) and 1260 RPH experiments on RG230-2 microarrays, and 941 human primary hepatocyte (HPH) *in vitro* experiments on HGU133-2 microarrays. MG430-2 CEL files for mouse liver experiments and mouse primary hepatocytes (MPH), and HGU133-2 CEL files for human liver and HepG2 microarray experiments were retrieved from the Gene Expression Omnibus [35] (GEO; http://www.ncbi.nlm.nih.gov/geo/) or Array Express [57] (https://www.ebi.ac.uk/arrayexpress/): GSE44783 (4, 15 day repeat-dose studies in male CD-1 mice for 14 drugs), GSE43977 (7 day repeat-dose studies in male C57BL/6J mice for 17 compounds), GSE28878, GSE51952 (72 drugs in HepG2 for 12, 24 or 48 hours exposure), GSE37031, GSE63067 (normal human liver), GSE57129, E-MEXP-2539, E-MEXP-2209, E-MEXP-2636 (19 drugs in MPH for 1 or 2 day exposure).

### Microarray data processing

Bioconductor version 2.13 was used throughout this work (http://www.bioconductor.org). CEL files were analyzed with the Affy package to produce MAS5 and RMA [58] probe set intensities. Normalization to controls was performed by taking the average MAS5 log intensity or average RMA log intensity across 2–5 biological replicates for the treatment group and subtracting the equivalent quantity for the control group.

### Probe set selection and ortholog mapping

In order to simplify comparisons across system and microarrays, we selected the highest intensity probe set per gene using expression levels in control samples for each combination of system and microarray. The Affymetrix-supplied annotations we used to map probe sets to Entrez gene IDs, retaining only probe sets that map to a single gene (version 33 for rat liver, RPH and HPH; version 35 for HepG2 and mouse liver. Downloaded from http://www.affymetrix.com/support/mas/index.affx?navMode=cat120004&aId=supportNav). The following data sources were used to select the highest intensity probe set for each gene from MAS5 normalized signals: Drug Matrix control arrays for rat liver and RPH, TG control arrays for HPH, GSE43977, GSE51885 and GSE44793 male controls for mouse liver (using both time points and 3 vehicles for GSE44793, and computing the average intensity across the three datasets giving each equal weight), E-MEXP-2209 for MPH (using all 4 time points for DMSO vehicle), GSE28878 for HepG2 (using all 3 time points for DMSO vehicle), GSE37031 and GSE63067 for normal human liver (excluded GSM1539891 from GSE63067 as it was an outlier when samples were clustered). We examined control intensity correlations of the individual GEO series before averaging and all exceed R-sq values of 0.88. While other strategies exist for selecting a representative probe set for each gene, intensity based selection leads to the best between-study consistency [59].

Throughout this work, we used the rat as reference organism and mapped human and mouse genes to rat using the RGD resource [60]. We downloaded the files GENES_RAT.txt and RGD_ORTHOLOGS.txt from ftp://rgd.mcw.edu/pub/data_release/ on 2015/01/15. This resulted in 19269 rat genes, mapped to 17459 human and 17366 mouse genes. Of these, 14078 / 15991 / 15691 are represented on the corresponding Affymetrix microarrays after the selection process described above. The complete mapping of rat genes, orthologs, selected Affymetrix probe sets and their intensities in control samples are provided in S5 Dataset.

### Gene-level analysis

Each experiment was described using a vector of features describing gene expression changes obtained by comparing treatment and control samples. A commonly-used analysis approach involves calculating fold change values for each gene represented on the microarray (and log-transformed to increase the normality of the distribution of fold change values). Since microarrays provide less reliable estimates of abundance for low-expressed genes [3, 23], we assembled a set of genes we describe as “rat liver-expressed genes” throughout this manuscript. Working from 20269 probe sets mapped unambiguously to one or more genes on the RG230-2 microarray, we identified a subset of 11884 probe sets with above median intensity (calculated on each microarray) in 10% or more of control arrays (expressed) or 10% of treatment arrays (inducible). Where a gene is represented by multiple probe sets among the set of 11884, we retained the highest intensity probe set as representative. This results in 9071 genes (identified in S5 Dataset) represented by one probe set per gene, or approximately 64% of the genes on the array. This result is in agreement with the approximately 60% of the genome estimated to be expressed in human liver [61]. Fold change values for each of the 9071 genes are described throughout this work as gene-level analysis.

There are many approaches for producing estimates of mRNA abundance from Affymetrix microarrays. Perhaps the most common “legacy” approach consists of conventional (manufacturer’s) probe sets with MAS5 normalization, using updated probe set annotations supplied by the manufacturer. A similar approach using conventional probe sets with RMA normalization is more widely applied in the recent literature. There is a large body of literature claiming advantages for one method vs. others, yet both MAS5 and RMA on conventional probe sets perform similarly in what is perhaps the most definitive comparison from large spike-in experiments (“MAS 5.0, RMA, GCRMA yielded between 84%-87% sensitivity at a 5% FDR”) [62]. More recently, probe sets have been redesigned by mapping underlying probes to modern genome builds (e.g. BrainArray probe sets), which has been shown to have significant effect for certain genes [63]. A comprehensive assessment from the SEQC initiative[64] demonstrates increased mutual information vs. RNA-seq for a newer analysis pipeline vs. the legacy approach. We continue to use the legacy approach and RG230-2 arrays for toxicogenomics applications to facilitate the interpretation of current studies in the context of thousands of legacy experiments where re-analysis is impractical. In order to evaluate the dependence of our results on the choice of normalization and probe set definitions, we repeated analyses in this work with updated methods.

As a representative “modern” approach for microarray data analysis, we selected RMA normalization and BrainArray version 19 probe sets designed against Entrez gene (http://brainarray.mbni.med.umich.edu/Brainarray/Database/CustomCDF/19.0.0/entrezg.asp), and mapped them to the conventional (i.e. manufacturer’s) probe sets via the Entrez gene ID. Of the 9071 liver-expressed genes using conventional probe sets, 8094 have a corresponding BrainArray probe set. For a given experiment, we compare logFC values for the 8094 genes using the modern and legacy approach, and calculate the Pearson correlation. We obtained minimum / 10^th^ percentile / median Pearson R values of 0.49 / 0.67 / 0.78 across the 654 DM rat liver experiments. When analyzing expression changes via modules or GSA, we find higher agreement for modules (minimum / 10^th^ percentile / median values of 0.63 / 0.84 / 0.92) and similar agreement for GSA (minimum / 10^th^ percentile / median values of 0.28 / 0.64 / 0.82). The median level of agreement is similar to that seen when analyzing the same samples with the same method across different sites in the MAQC-I initiative (S8 Table).

We repeated all cross-source and cross-model comparisons with expression data from the modern processing approach. Here, we focus on three comparisons within TG because they represent trends based on hundreds of drugs (S10 Table), although the same behaviors are seen for the other comparisons. For genes and modules, concordance as assessed via Pearson R or the overlap metric increases or decreases slightly when using modern processing, with increases limited to ΔR ≤ 0.08 and Δ overlap ≤ 4%; success at self-identification decreases slightly. The improvement is greater for GSA concordance (ΔR ≤ 0.16 and Δ overlap ≤12), although as observed for the other methods performance in self-identification mostly decreases (and GSA was already the worst performing of 3 methods for self-identification when using legacy processing). Versions of Figs 2, 4 and S4 created using modern processing are given in S6, S7 and S8 Figs, respectively, and the full data is available in S2 Dataset. In summary, the results presented in this work are minimally impacted by methods used for microarray data processing. Unless otherwise noted, results in this work use MAS5-normalized gene expression results.

### Gene set analysis (GSA)

Identification of pathways enriched among differentially expressed genes is frequently performed to simplify interpretation of results from expression profiling studies [65]. The popular GSEA algorithm [5] is perhaps the current “gold-standard” approach, and unlike classic enrichment tests that require definition of what constitutes a differentially-expressed gene (e.g. fold change > 1.5 and p-value < 0.05), GSEA and related gene set analysis (GSA) algorithms operate on gene-level statistics and avoid the need for arbitrary cutoffs. Here we use the PAGE algorithm [36] implemented in the Piano package since its run time is substantially less than GSEA and provides similar results [65]. The syntax used in this work is runGSA(genes,gsc = gsc,geneSetStat = "page",gsSizeLim = c(3,5000),signifMethod = "nullDist", adjMethod = "BH"). We use only one probe set per gene, selected based on intensity in controls (see above). To conform with standard GSA analysis, we use all genes on the microarray, not only the 9071 liver-expressed genes.

Gene sets for GSA analysis were selected from two different sources. The canonical pathway collections (CP) from MSigDB version 4 [5] were obtained from http://www.broadinstitute.org/gsea/downloads.jsp. The provided human Entrez geneIDs were validated and updated if necessary using the gene_info and gene_history files from NCBI (ftp://ftp.ncbi.nlm.nih.gov/gene/DATA/). The resulting human Entrez gene IDs were converted to rat gene IDs using the orthology map described above, resulting in 1320 CP gene sets ranging in size from 4 to 823 rat genes. In total, 59497 gene vs. gene set pairs were obtained involving 7572 unique rat genes. For gene ontology (GO) terms, we obtained the GO ontology from http://purl.obolibrary.org/obo/go/go-basic.obo, the official RGD GO to gene association for rat from http://geneontology.org/gene-associations/gene_association.rgd.gz, using all evidence codes. The latter provides identifiers for RGD and UniProtKB gene and protein identifiers. We used the above-mentioned GENES_RAT.txt file for RGD gene ID to Entrez gene ID conversion, and the UniProt mappings to Entrez gene from ftp://ftp.uniprot.org/pub/databases/uniprot/current_release/knowledgebase/idmapping/by_organism/RAT_10116_idmapping.dat.gz. We produced the full GO to gene association for the mappings in gene_association.rgd by propagating up in the GO ontology using a perl script (“is_a”, “part_of”, “positively_regulates”, “negatively_regulates” and “regulates” associations). We retained GO terms of the “biological process” and “cellular component” types containing between 3 and 5000 rat genes. This resulted in 10261 GO terms and 1089835 GO term vs. gene pairs involving a total of 15408 rat genes.

As done for the gene-level analysis, we described the transcriptional effects of drug treatment as a vector of Z-scores from PAGE, using one Z-score for each gene set (the Z-score is the statistic indicating the degree of enrichment for a given gene set among the most induced or repressed genes). Gene sets may contain genes that are not co-expressed (either in liver or any tissue or cell), and hence will not be identified as enriched among differentially expressed genes in liver experiments. In order to reduce the sparseness of this vector, we identified a subset of 1719 gene sets from the full set of 10261 GO and 1320 CP gene sets) that have BH-adjusted p ≤ 0.01 in 1% of TG rat liver experiments or more (≥35 experiments). We added back 121 gene sets falling below this threshold, but pertaining to processes of relevance to drug-induced liver injury (inflammation, oxidative stress, endoplasmic reticulum stress, ubiquitination, apoptosis, etc.) The complete set of 1840 gene sets used for GSA analysis is provided in S6 Dataset.

### Co-expression network analysis

We used the WGCNA [37] package in Bioconductor to derived co-expression networks using the 654 DM rat liver experiments (henceforth “training” data) and 9071 liver-expressed genes (i.e. a matrix with 9071 rows and 654 columns, containing log10 fold-change value from MAS5 intensities). We used the standard power-law plotting tool in WGCNA to set the soft-power parameter to 8. Modules were unsigned (i.e. can contain both induced and repressed genes; TOM = TOMsimilarity(adjacency,TOMType = "unsigned")). The gene dendrogram was built using average-linkage hierarchical clustering (geneTree = flashClust(as.dist(dissTOM), method = "average")), and the modules created with the dynamic branch cut algorithm (cutreeDynamic(dendro = geneTree, distM = 1-TOM, deepSplit = 4, pamRespectsDendro = FALSE, minClusterSize = 5)). This yielded 354 modules containing a total of 8014 genes. An additional set of 61 merged modules were defined by grouping together 131 modules having Pearson correlation of their eigengenes (see below) R ≥ 0.8 across the training data. We describe the 61 merged + 223 unmerged (i.e. those having max R < 0.8 vs. the other 353 modules) as “base” modules.

For any given experiment, each module is described by one module score, or “eigengene”, using principal components analysis (PCA) performed on the training data for its component genes. Prior to PCA, log10 fold change values (logFC) are Z-scored using the average and standard deviation of each gene’s logFC values across the training experiments. The fraction of variance of the underlying Z-scored logFC values explained by the first PC has a quasi-normal distribution, with average / standard deviation of 0.48 ± 0.09 across the 415 modules. Modules containing more genes have lower variation explained by the first PC (Spearman ρ = -0.67). The standard deviation of raw module scores, calculated using the 654 DM rat liver experiments, varies by module and is explained largely by the number of genes in the module (ρ = 0.97). In order to simplify interpretation, a final module score is obtained by dividing the raw module score by its standard deviation within the training data. As such, a module score for an experiment reflects that drug’s effect on the module measured in standard deviations across the training data. Throughout this work, we used the average absolute eigengene score (avg. abs. EG) as a measure of overall transcriptional activity. This quantity is calculated on the 284 base modules only in order to minimize redundancy among the modules.

The WGCNA literature is dominated by sample-level analyses, i.e. human samples, cell lines, etc, where gene-level intensities are centered across samples by virtue of microarray or RNA-seq normalization algorithms. When compared to a control group, many genes have non-zero logFC. This is not surprising given the expected preponderance of xenobiotic and other responses in a dataset like DM, where doses selected were often high in order to generate histology findings in rat liver. For example, the Abcc3 subunit of the MRP drug transporter (probe set 1369698_at) has an average fold change of 3.2. Centering the data for such genes causes experiments where logFC is near zero to take on (usually small) positive or negative values after subtracting average logFC across the training data. Since modules contain co-expressed genes, experiments where the component genes have logFC close to zero can appear as induced or repressed. For this reason, we center and scale for determining a gene’s weight in the module at the module building stage, but we simply scale logFC values by the standard deviation of the gene and multiply by the gene’s PCA loading when scoring experiments. The process is illustrated for an example module in S7 Dataset. Module definitions, gene weights and average / standard deviation of logFC across DM and TG are given in S8 Dataset.

Preservation analysis serves to evaluate the relevance of a module in other systems [66]. We used the module Preservation function in the WGCNA package to calculate the Z-summary preservation score in TG rat liver and TG RPH. The score encompasses a number of metrics for assessing the properties of a module, and is normalized vs. the equivalent scores for modules consisting of random gene selections. We used 200 random gene selections to calculate module preservation statistics.

WGCNA analysis can be regarded as producing gene sets having different weights in different systems (or biological “contexts”), whereas GSA and related methods give all genes equal and non-varying weight. As such, parameters determined in one system will not be meaningful in all systems. Scoring of modules requires transforming logFC values to Z-scores using a gene’s stdev of logFC (i.e. scaling on gene variability), and applying the weight of that gene in a given module. Both scaling and weights will vary across systems, giving rise to several possible choices when applying modules derived from DM rat liver to other systems: 1) assume that logFC stdev and gene weights from the derivation system apply in the new system, 2) use logFC stdev in the new system to Z-score logFC values, but use gene weights from the derivation system, or 3) use logFC stdev from the new system and re-calibrate the weight of genes within modules using PCA. For small datasets, like the mouse data discussed in this work, re-deriving module weights and logFC statistics would be challenging and 1) constitutes the only practical choice. The concordance data presented in this work uses DM liver weights for all systems, with variation in data source selected for scaling: DM rat liver for all rodent liver results, DM RPH for all RPH results, and TG HPH for HPH and HepG2 results. We investigated the impact of scaling and re-calibration post-hoc by comparing module scores for various scenarios (S11 Table). The impact of using different data sources for scaling is small, in part due to high correlation of gene variability of the 9071 liver-expressed genes across sources (S12 Table). Recalibration from DM liver weights to RPH or HPH weights has a larger effect, but this is markedly reduced when focusing only on modules that are preserved in the new system (Z-summary ≥ 3). This result is intuitive: the preservation score of a module measures the degree of co-expression of its member genes in the new system, and having high co-expression in both systems results in similar gene weights when calibration is performed in either system. Thus, it appears practical to apply gene weights across related systems (in this case, systems consisting primarily of hepatocytes), and evaluate preservation of the module in the new system where sufficient perturbation data allow. Similarly, we investigated the impact of using RMA-normalized DM liver data (with conventional probe sets) for module calibration and found minimal effects on scoring of experiments (minimum / 10^th^ percentile / median Pearson R values of 0.82 / 0.94 / 0.98 comparing MAS5 and RMA scores for 654 DM liver experiments).

When WGCNA analysis was performed at the onset of this work, we selected the probe set with the highest standard deviation of logFC values across the rat liver DM dataset as the representative for a given gene (i.e. the most variable probe set for the gene). This was subsequent to application of the intensity-based filter: a probe set still needed to be above median intensity in 10% of control or treatment samples. The combination of intensity and variability-based selection was impractical for systems where we have few samples (i.e. 10% of samples might represent a few samples for small datasets). When using DM rat liver data, the probe set selected for the 9071 liver expressed genes based on maximal intensity differs from that selected as most variable for 1670 genes. For application of the rat liver-derived modules to other systems, we adopted the recommendation from [59] in selecting as representative probe set that with maximal intensity. The eigengene scores used in this work use the probe set selected by variability for scoring rat liver and RPH, and intensity-selected probe sets for mouse and human samples. We re-calibrated the original DM rat liver modules using the highest intensity probe set for these 1670 genes, to obtain gene weights that correspond to the higher intensity probe set. Comparison of module scores using the original variability-based and intensity-based selection across the 654 rat liver DM experiments gives minimum / 10^th^ percentile / median Pearson R values of 0.83/0.94/0.98. The variability-based and intensity-based probe set selections, along with weights required for module scoring with either selection are provided in S8 Dataset. Overall, module eigengenes are robust to changes in genes / probe sets selected. We re-derived the modules on the DM rat liver data using the complete set of 14078 genes, selecting the highest intensity probe set for each gene and applying no intensity cutoff to eliminate low expression genes, obtaining 381 modules that contain 8639 genes in total (vs. the 8014 contained in modules on our original build). When calculating the pairwise correlation between original and new modules across the 654 DM rat liver experiments, 374 of 415 original modules have Pearson R ≥0.7 vs. a new module, and 308 have Pearson R ≥ 0.8.

### Calculation of percent overlap

Percent overlap is a qualitative metric popularized by the MAQC initiative[3, 23, 24] which simply reports what percentage of the top-ranked features overlap between two experiments. We illustrate its implementation in this work using the 9071 liver-expressed genes, with equivalent applications to GSA or modules at the same 5% threshold. For each experiment, the top 5% of genes are identified, with no consideration of direction (induced or repressed), giving 454 genes. When comparing two experiments, we identify the number of genes in common between both lists of 454 genes and changing in the same direction in both experiments. This value is divided by 454 and reported as a percentage.

### Comparison of expression concordance to random experiment pairs

We examined the distribution of average absolute eigengene scores (avg. abs. EG) for 78,226 pairs of TG rat liver experiments and 10,008 TG RPH experiments, and divided the range into 7 intervals: <0.2, 0.2–0.3, 0.3–0.4, 0.4–0.5, 0.5–0.6, 0.6–0.8 and ≥ 0.8. For each of 3 sets of gene expression features (genes, gene sets and modules) and two metrics (Pearson R and percent overlap), we generated 1000 random pairs of experiments involving different drugs falling into each avg. abs. EG range. When evaluating the concordance for a pair of experiments involving the same drug, we report the proportion of the 1000 random pairs with equal or greater concordance on the same feature and metric and falling in the same avg. abs. EG range. When defining success at ‘self-identification’, we require fewer than 5% of random pairs to exceed the level of concordance seen for the pair in question.

### Euclidian distance as a metric for gene-expression profile similarity

Several metrics are available for quantifying the similarity of two gene expression profiles. In this work we have selected Pearson R and percent overlap because we consider them to be intuitive metrics when comparing two conditions (plotting genes’ logFC via scatter plots, for example). The similarity of expression profiles can be quantified with the Euclidian distance, and this represents a common choice when performing principal components analysis and clustering on gene expression profiles [64]. Identical gene expression profiles will have a Euclidian distance of 0 and Pearson correlation of 1. We calculated the Euclidian distances between 78,226 pairs of rat liver experiments and 10,008 pairs of RPH experiments from TG, and compared them to the Pearson distance (1 –Pearson R). Euclidian and Pearson distances are uncorrelated for genes and modules across both systems (R < 0.17), and modestly correlated for GSA (R = 0.53 and 0.63 for rat liver and RPH, respectively). Euclidian distance effectively regards a common absence of differentially expressed features as evidence of similarity, giving equal importance to the usually far larger number of non-differentially expressed features compared to Pearson R or overlap metrics. We evaluated the performance of Euclidian distance in self-identification for all comparisons depicted in Figs 2, 4 and S4. Compared to the Pearson R (S9 Fig) and overlap metrics (data in S2 Dataset), Euclidian distance performs similarly for GSA and notably worse for genes and modules. Due to its lower performance and less intuitive characteristics (“your molecule is similar to drug X because neither of have a significant effect on the liver transcriptome”), we do not advocate its application for CMap-type applications. Results for all comparisons, using both legacy and modern array normalization methods, are provided in S2 Dataset.

## Supporting Information

S1 Fig Comparison of concordance metrics across 78226 rat liver and 10008 RPH experiment pairs from TG-GATEs involving the same drug. The percentage overlap among the most differentially expressed 5% of features is compared to the Pearson correlation coefficient and the overlap metric using the top 2.5% or 10% of differentially expressed features. Transcriptional effects of drugs are determined at A) gene-level, B) gene set analysis (GSA) and c) co-expression modules. Points are colored by non-parametric density estimation, and are jittered for GSA and modules for the overlap metrics due to the limited number of discrete values that occur (0/21, 1/21, 2/21, … 21/21 modules overlap among top 5%).
(TIF)

S2 Fig Relationship between percent of genes differentially expressed and average absolute eigengene score (avg. abs. EG). Experiments from rat liver and rat primary hepatocytes, taken from Drug Matrix and TG-GATEs were used to compare two measures of overall transcriptional effects of treatment. Points are colored by non-parametric density estimation. The subset of 8014 genes included in co-expression modules were used to calculate percentage of differentially expressed genes (fold change ≥ 1.5 and non-adjusted limma p-value ≤ 0.05). Other definitions of differential expression (fold change and/or p-value cutoffs) give similar results (results not shown).
(TIF)

S3 Fig Relationship between concordance and probability of self-identification success. Concordance using A) the Pearson R metric and B) overlap metric is evaluated for all pairs of experiments involving the same drug, and assigned to a range on the X-axis. The Y-axis denotes the probability that a given level of concordance is exceeded by fewer than 5% of random pairs involving different drugs, averaged for all pairs in the range. Each panel describes the relationship for experiment comparisons between sources or systems (columns) and different levels of transcriptional activity (rows).
(TIF)

S4 Fig Concordance of drug-induced effects in rat primary hepatocytes. Pairs of experiments involving the same drug are compared via the Pearson R and percent overlap metrics, and average concordance is calculated between A) *TG RPH* vs. itself (within-source), B) *TG RPH* vs. DM RPH (cross-source) and C) *TG rat liver* vs. DM RPH (cross-source and cross-system). Concordance is shown additively for 3 levels of constraint on dose and time differences: experiments at the same time point and doses within 5 fold (pink), time within 2 fold and doses within 10 fold (blue), no constraint on doses or time (green). For each pair of data sources, experiments from one source are compared to all those from the reference source (italicized) and the most concordant experiment selected within the level of constraint. Because concordance can only improve upon removing dose and time constraints, results are shown additively with the total bar height denoting the concordance achieved with no constraints. Numeric values represent the probability that the given level of concordance exceeds that of random pairs involving different drugs for the most stringent level considered (pink bars). Experiment pairs are separated into 3 ranges based on the level of transcriptional activity for the least perturbing of two treatments (lowest quartile have avg. abs. EG ≤ 0.28, highest quartile have avg. abs. EG > 0.46; thresholds selected using 3528 TG rat liver experiments).
(TIF)

S5 Fig Alternate representation of concordance for genes, GSA and modules by quartiles of transcriptional activity (avg. abs. EG). Comparisons within and between sources and models are shown using separate lines as indicated in the legend. Data correspond to comparisons with no dose or time constraints (green bars) in Figs 2, 4 and S4.
(TIF)

S6 Fig Concordance of drug-induced effects in rat and mouse liver using modern array processing. See Fig 2 caption for details, which this figure replicates exactly except for array processing.
(TIF)

S7 Fig Concordance of drug-induced effects in cultured hepatocytes using modern array processing. See Fig 4 caption for details, which this figure replicates exactly except for array processing.
(TIF)

S8 Fig Concordance of drug-induced effects in rat primary hepatocytes using modern array processing. See S4 Fig caption for details, which this figure replicates exactly except for array processing.
(TIF)

S9 Fig Comparison of self-identification success probability when using Pearson R vs. Euclidian distance as metric. The solid line indicates equal performance, while dashed lines indicate the interval [0.1, -0.1] around the solid line (i.e. an arbitrary definition of “similar performance”). For each axis, increasing self-ID success towards 1 denotes increasing performance.
(TIF)

S1 Table Concordance of TG-GATEs experiment pairs involving the same drug as a function of dose differences, time differences and average absolute eigengene score. (DOCX)

S2 Table Relationship between overall transcriptional activity and concordance of random experiment pairs involving different drugs from TG rat liver. (DOCX)

S3 Table Concordance between rat liver and rat primary hepatocytes (RPH) for select drug classes from TG using co-expression modules preserved in RPH. (DOCX)

S4 Table Concordance of LC50 values obtained by viability assays using cultured rat primary hepatocytes and human HepG2 cells. (DOCX)

S5 Table Concordance of mouse primary hepatocytes vs. rat primary hepatocytes and mouse liver. (DOCX)

S6 Table Pearson correlation between expression levels of genes in control samples for MAS5 and RMA normalized intensities. (DOCX)

S7 Table Summary of clinical chemistry and histology findings for 29 day methapyrilene and 1 day bortezomib treated rats. (DOCX)

S8 Table Re-analysis of MAQC-I Toxicogenomics results in rat liver for three treatments and comparison to TG rat liver within-source concordance. (DOCX)

S9 Table Probability of success for self-identification when comparing expression profiles of drugs across models and sources. (DOCX)

S10 Table Comparing concordance and probability of success in self-identification within TG-GATEs data for legacy and modern Affymetrix array processing. (DOCX)

S11 Table Concordance of module scores using gene variability scaling and module weight calibration from DM rat liver vs. scores obtained by rescaling or recalibrating modules in other systems. (DOCX)

S12 Table Spearman rho correlation of logFC standard deviation for 9071 liver-expressed genes (8349 for HPH due to missing orthologs) across various systems. (DOCX)

S1 Dataset Compilation of experiments used for concordance analysis. (XLS)

S2 Dataset Concordance results by system pair, method and quartile of transcriptional activity. (XLS)

S3 Dataset Pairwise similarity of expression profiles using modules and 9071 liver expressed genes. (XLS)

S4 Dataset Parametric enrichment of gene set enrichment analysis on rat primary hepatocyte timecourse experiment. (XLS)

S5 Dataset Rat-mouse-human ortholog mappings and assignment of microarray probe sets with their corresponding intensity in controls. (XLS)

S6 Dataset Gene sets used for PAGE analysis. (XLS)

S7 Dataset Example for scoring one module. (XLS)

S8 Dataset Module definitions and calibration results in DM and TG rat liver. (XLS)
