# Supplementary figures and images for: Assessing Concordance of Drug-Induced Transcriptional Response in Rodent Liver and Cultured Hepatocytes
Source: PLoS Comput Biol. 2016 Mar 30;12(3):e1004847. doi: 10.1371/journal.pcbi.1004847 (PMC4814051; doi:10.1371/journal.pcbi.1004847)

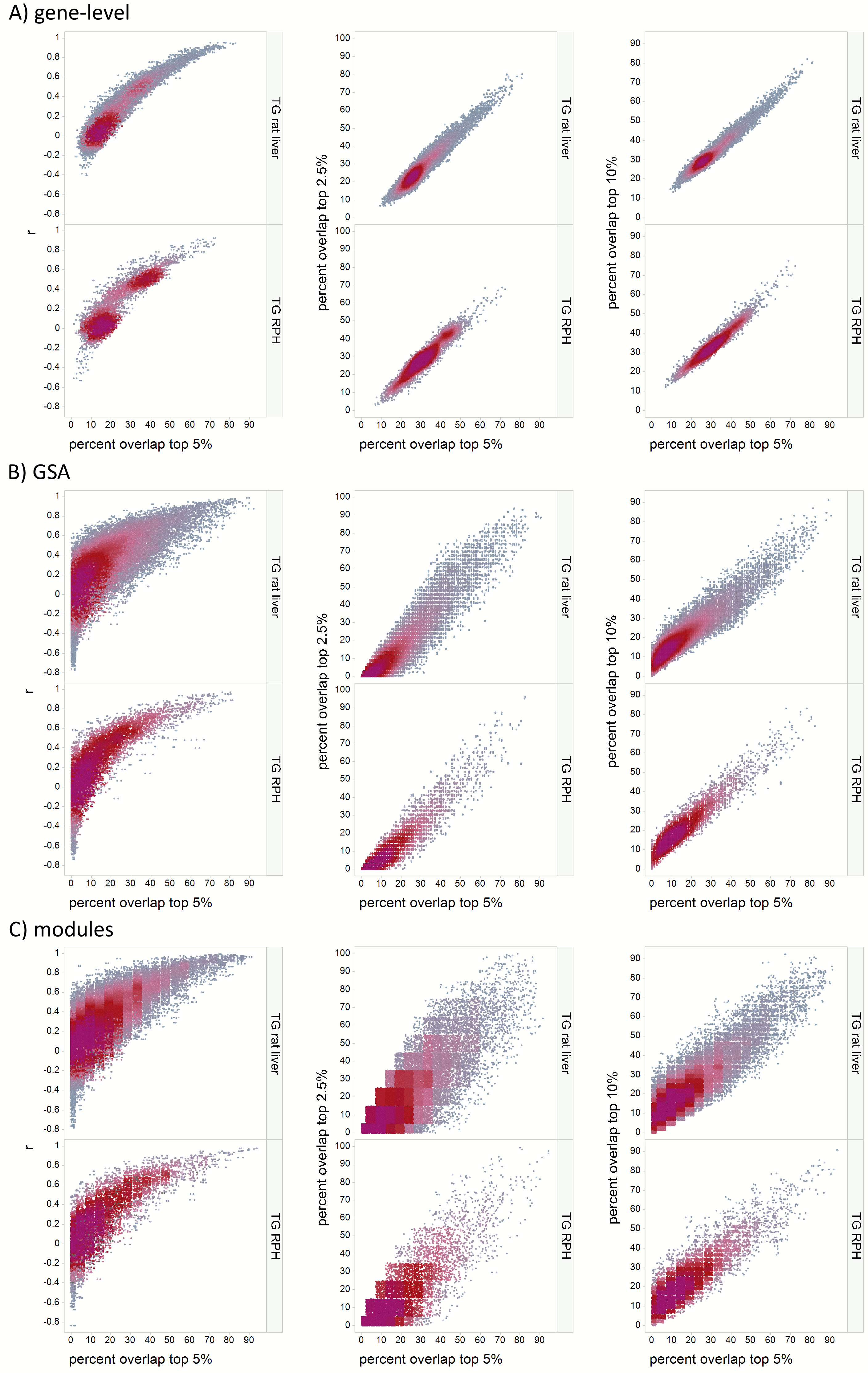

Supplement: S1 Fig — The percentage overlap among the most differentially expressed 5% of features is compared to the Pearson correlation coefficient and the overlap metric using the top 2.5% or 10% of differentially expressed features. Transcriptional effects of drugs are determined at A) gene-level, B) gene set analysis (GSA) and c) co-expression modules. Points are colored by non-parametric density estimation, and are jittered for GSA and modules for the overlap metrics due to the limited number of discrete values that occur (0/21, 1/21, 2/21, … 21/21 modules overlap among top 5%). (TIF) [file pcbi.1004847.s001.tif]

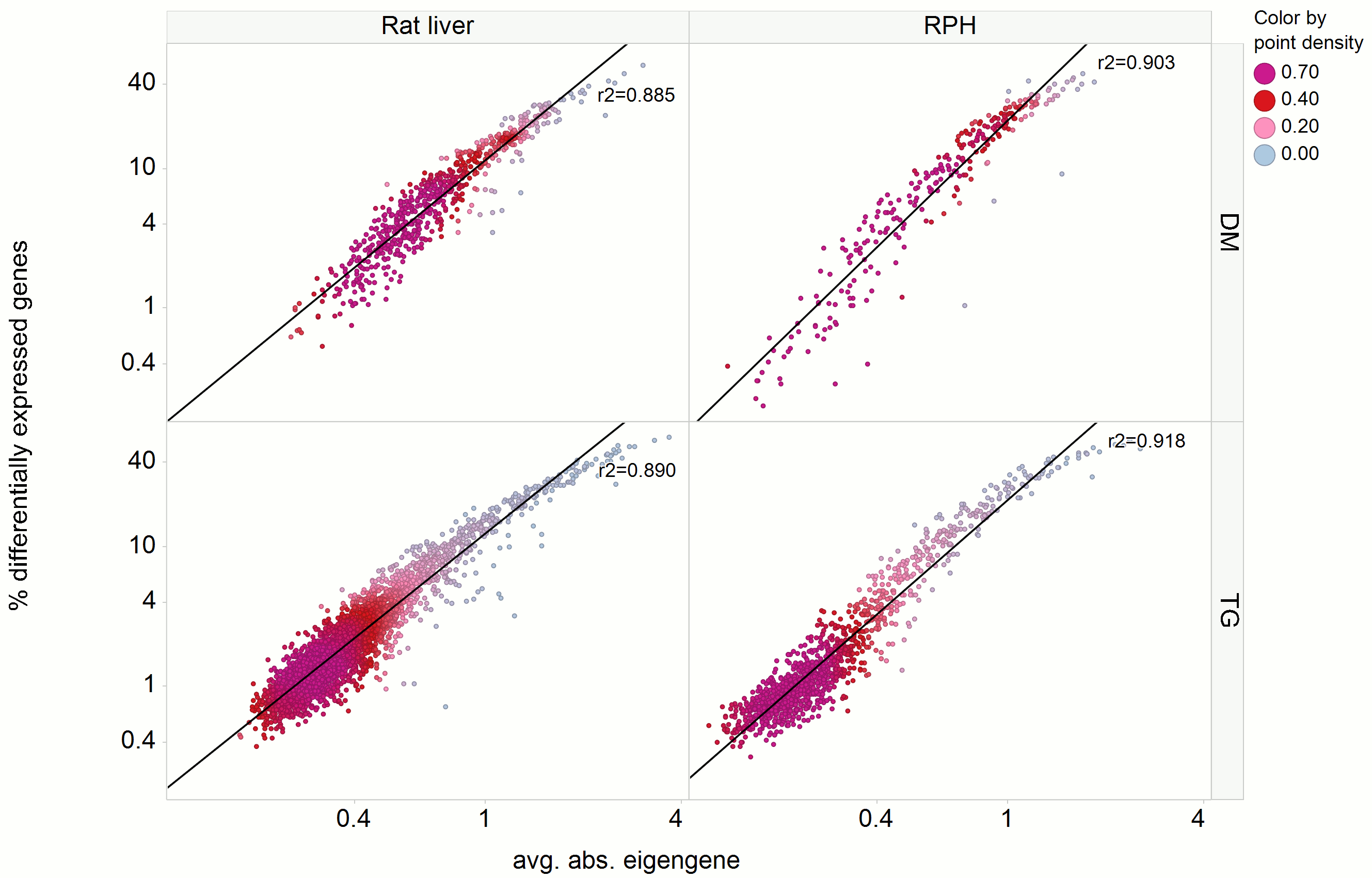

Supplement: S2 Fig — Experiments from rat liver and rat primary hepatocytes, taken from Drug Matrix and TG-GATEs were used to compare two measures of overall transcriptional effects of treatment. Points are colored by non-parametric density estimation. The subset of 8014 genes included in co-expression modules were used to calculate percentage of differentially expressed genes (fold change ≥ 1.5 and non-adjusted limma p-value ≤ 0.05). Other definitions of differential expression (fold change and/or p-value cutoffs) give similar results (results not shown). (TIF) [file pcbi.1004847.s002.tif]

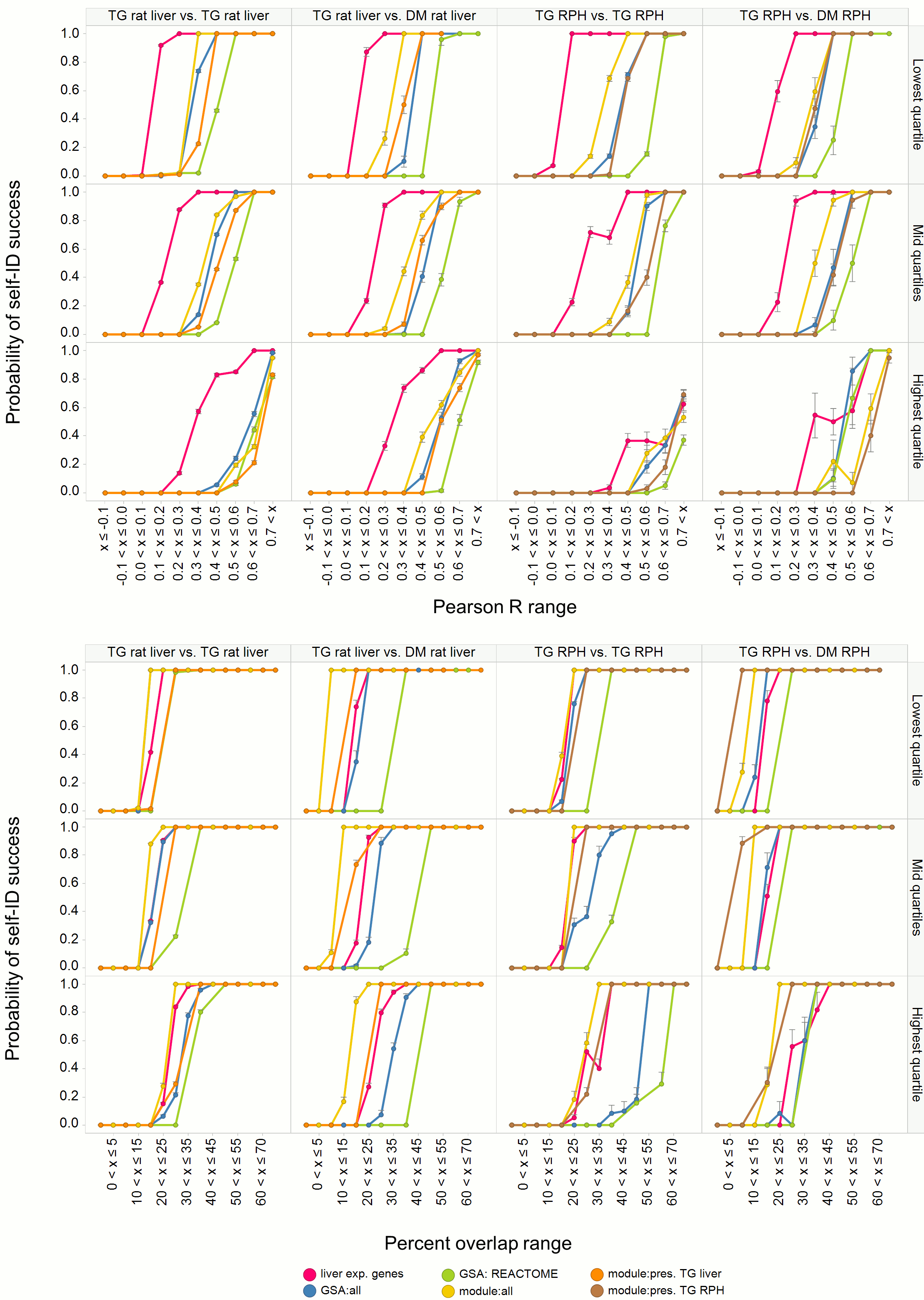

Supplement: S3 Fig — Concordance using A) the Pearson R metric and B) overlap metric is evaluated for all pairs of experiments involving the same drug, and assigned to a range on the X-axis. The Y-axis denotes the probability that a given level of concordance is exceeded by fewer than 5% of random pairs involving different drugs, averaged for all pairs in the range. Each panel describes the relationship for experiment comparisons between sources or systems (columns) and different levels of transcriptional activity (rows). (TIF) [file pcbi.1004847.s003.tif]

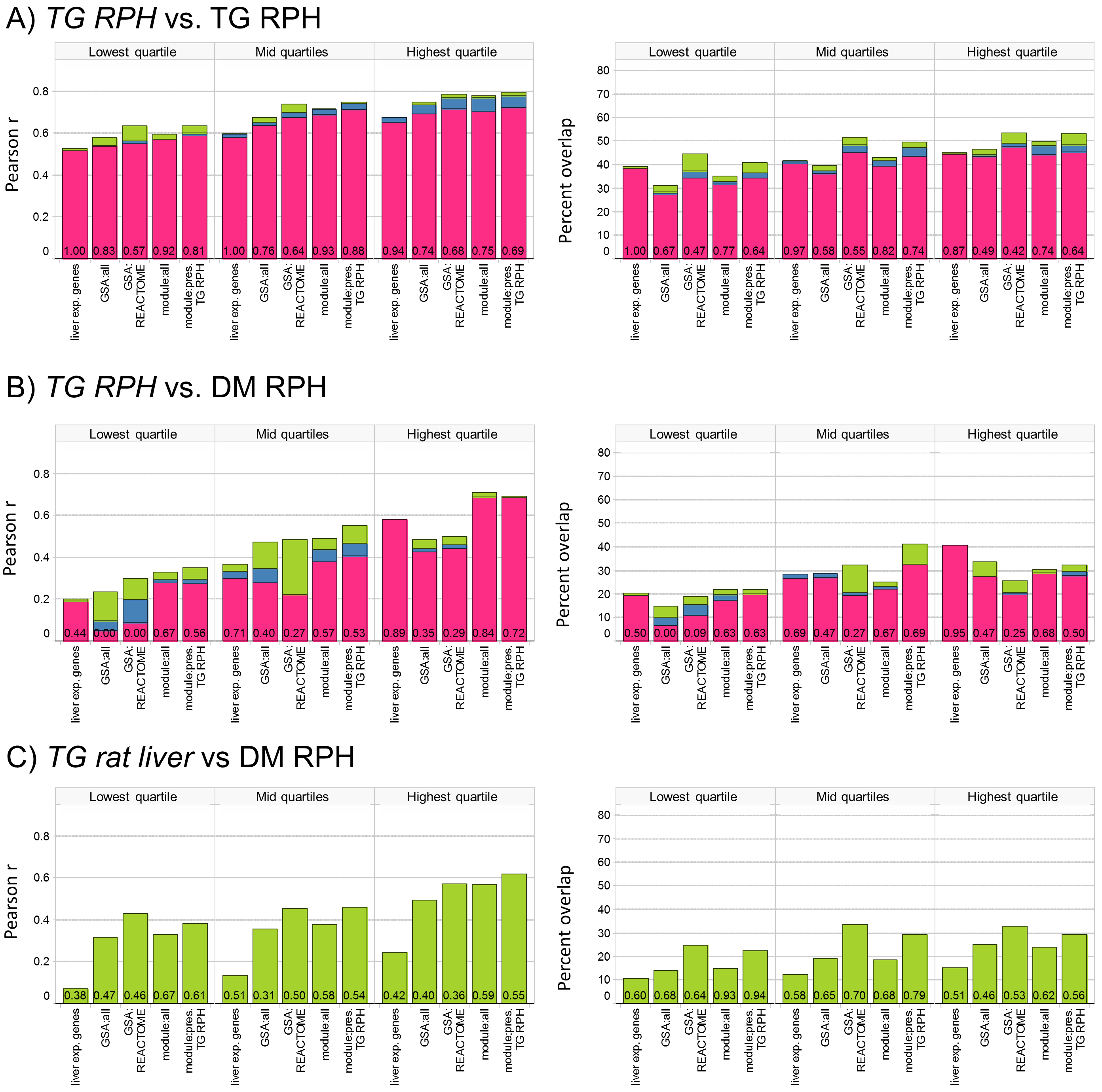

Supplement: S4 Fig — Pairs of experiments involving the same drug are compared via the Pearson R and percent overlap metrics, and average concordance is calculated between A) TG RPH vs. itself (within-source), B) TG RPH vs. DM RPH (cross-source) and C) TG rat liver vs. DM RPH (cross-source and cross-system). Concordance is shown additively for 3 levels of constraint on dose and time differences: experiments at the same time point and doses within 5 fold (pink), time within 2 fold and doses within 10 fold (blue), no constraint on doses or time (green). For each pair of data sources, experiments from one source are compared to all those from the reference source (italicized) and the most concordant experiment selected within the level of constraint. Because concordance can only improve upon removing dose and time constraints, results are shown additively with the total bar height denoting the concordance achieved with no constraints. Numeric values represent the probability that the given level of concordance exceeds that of random pairs involving different drugs for the most stringent level considered (pink bars). Experiment pairs are separated into 3 ranges based on the level of transcriptional activity for the least perturbing of two treatments (lowest quartile have avg. abs. EG ≤ 0.28, highest quartile have avg. abs. EG > 0.46; thresholds selected using 3528 TG rat liver experiments). (TIF) [file pcbi.1004847.s004.tif]

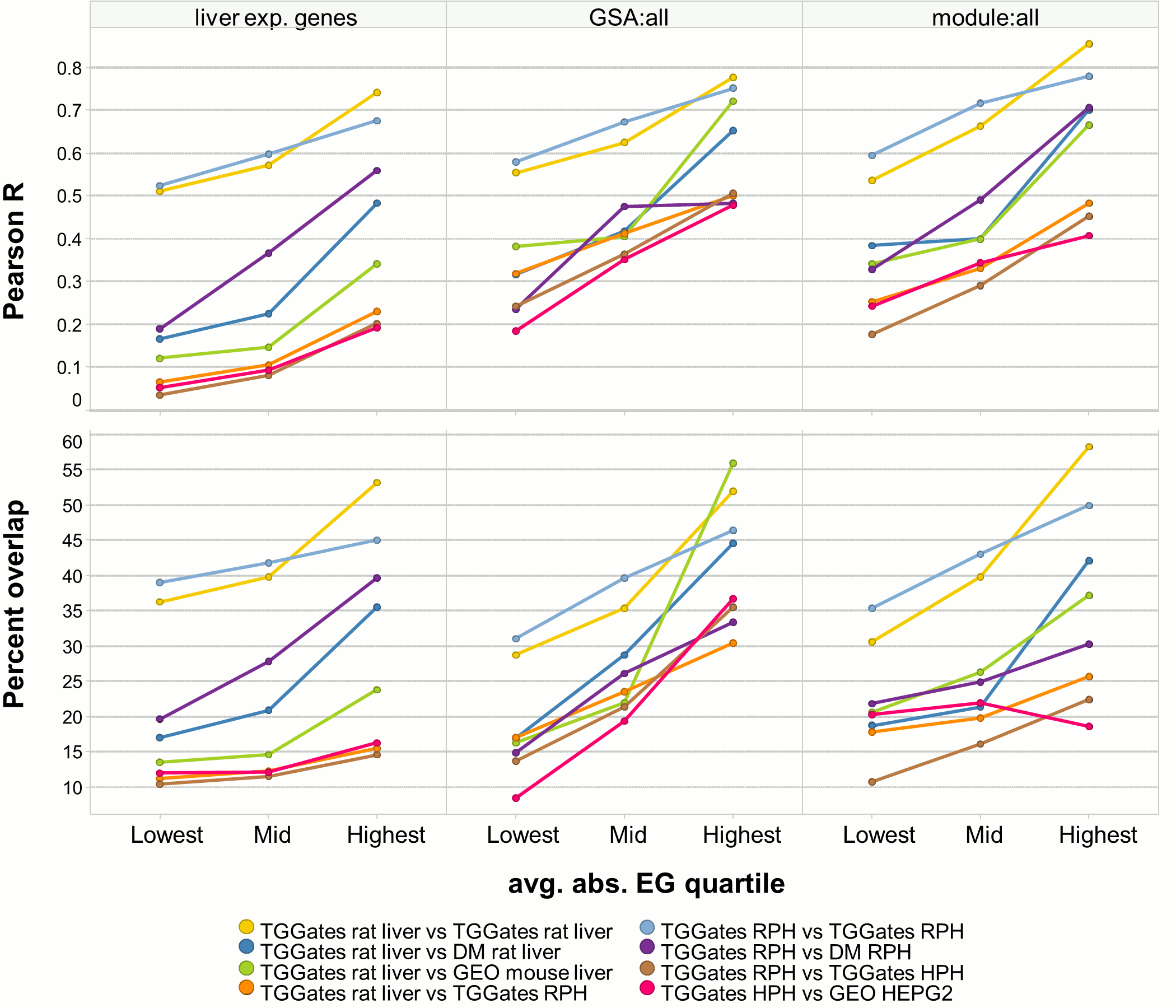

Supplement: S5 Fig — Comparisons within and between sources and models are shown using separate lines as indicated in the legend. Data correspond to comparisons with no dose or time constraints (green bars) in Figs 2, 4 and S4. (TIF) [file pcbi.1004847.s005.tif]

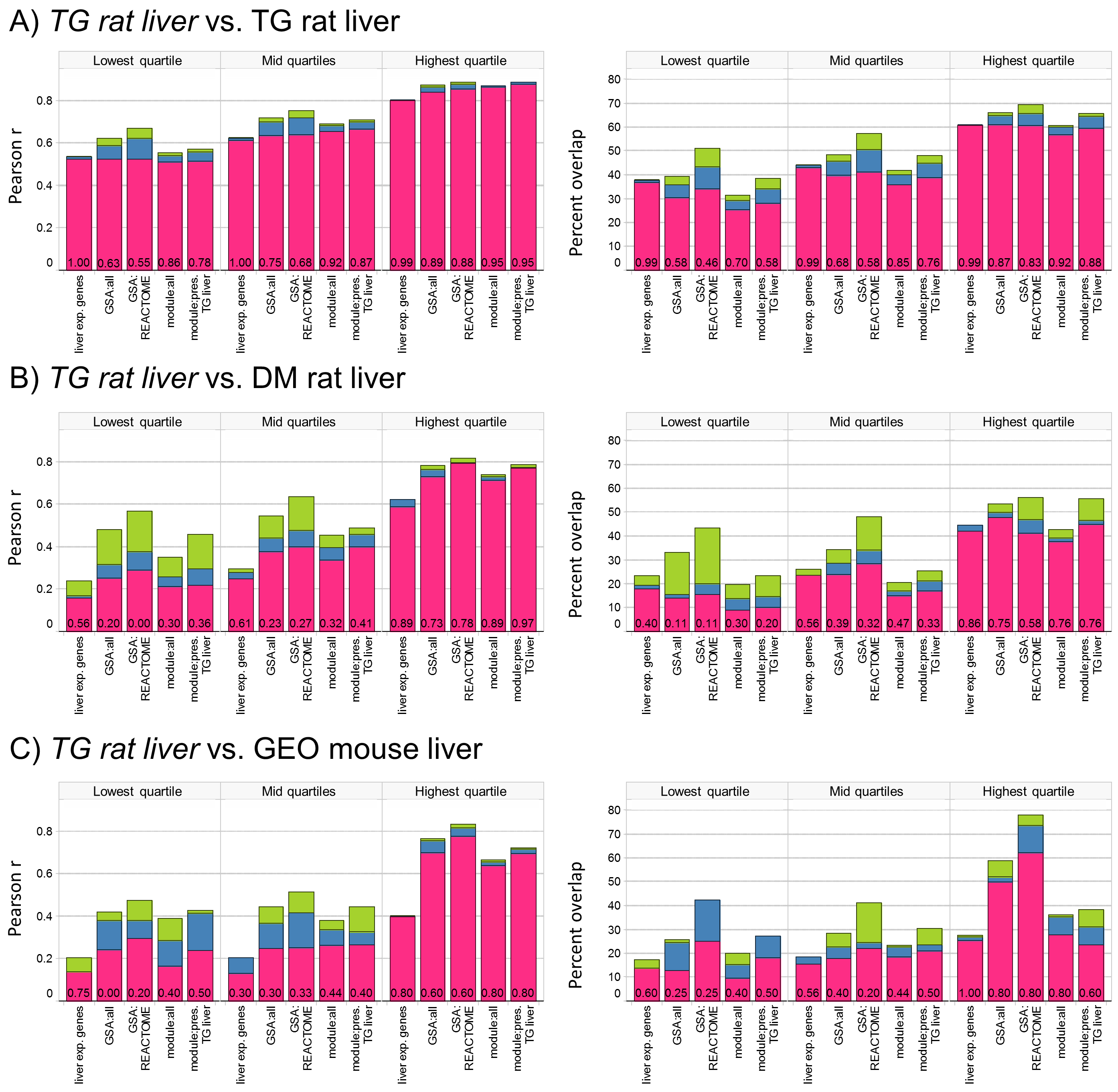

Supplement: S6 Fig — See Fig 2 caption for details, which this figure replicates exactly except for array processing. (TIF) [file pcbi.1004847.s006.tif]

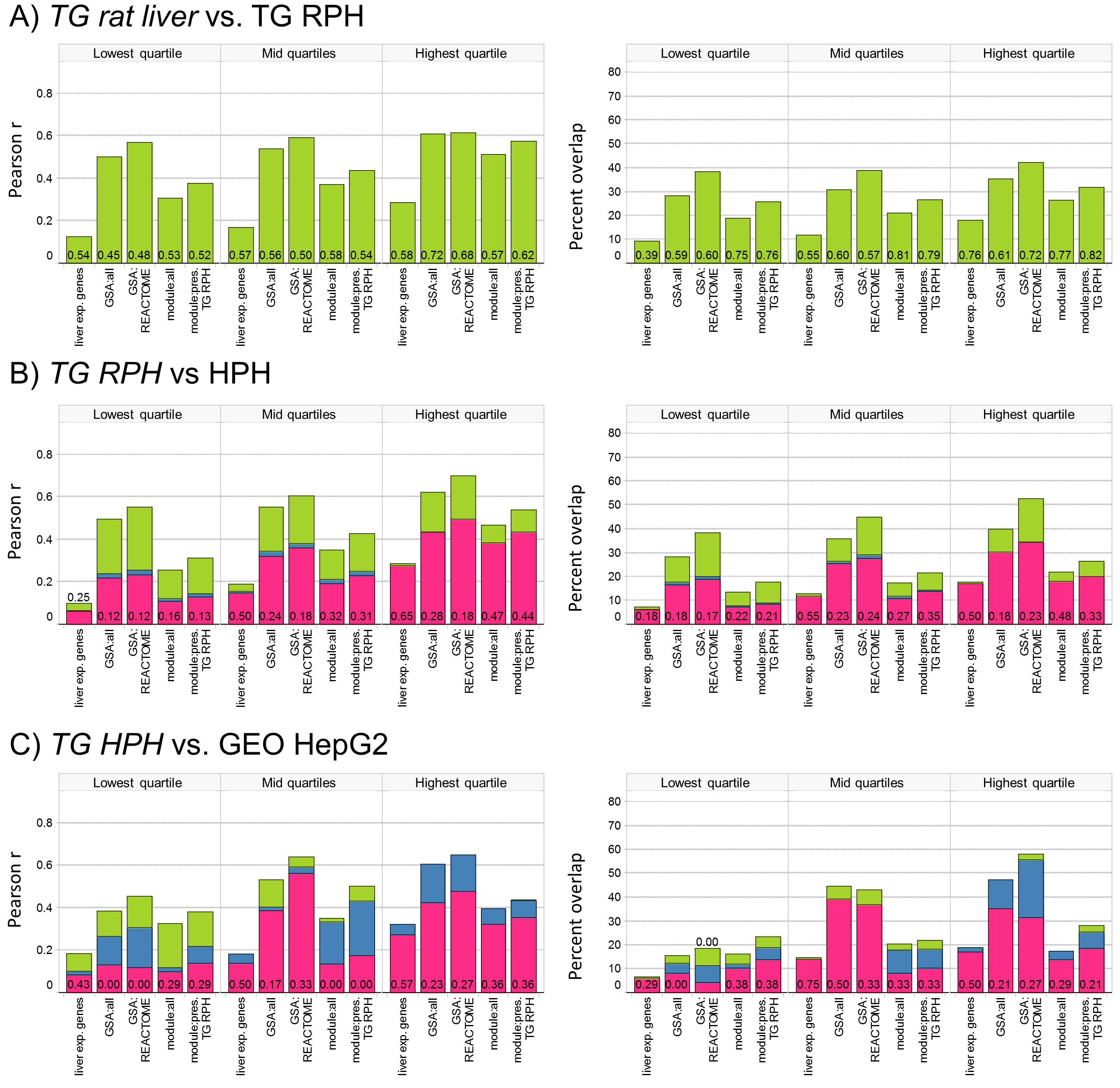

Supplement: S7 Fig — See Fig 4 caption for details, which this figure replicates exactly except for array processing. (TIF) [file pcbi.1004847.s007.tif]

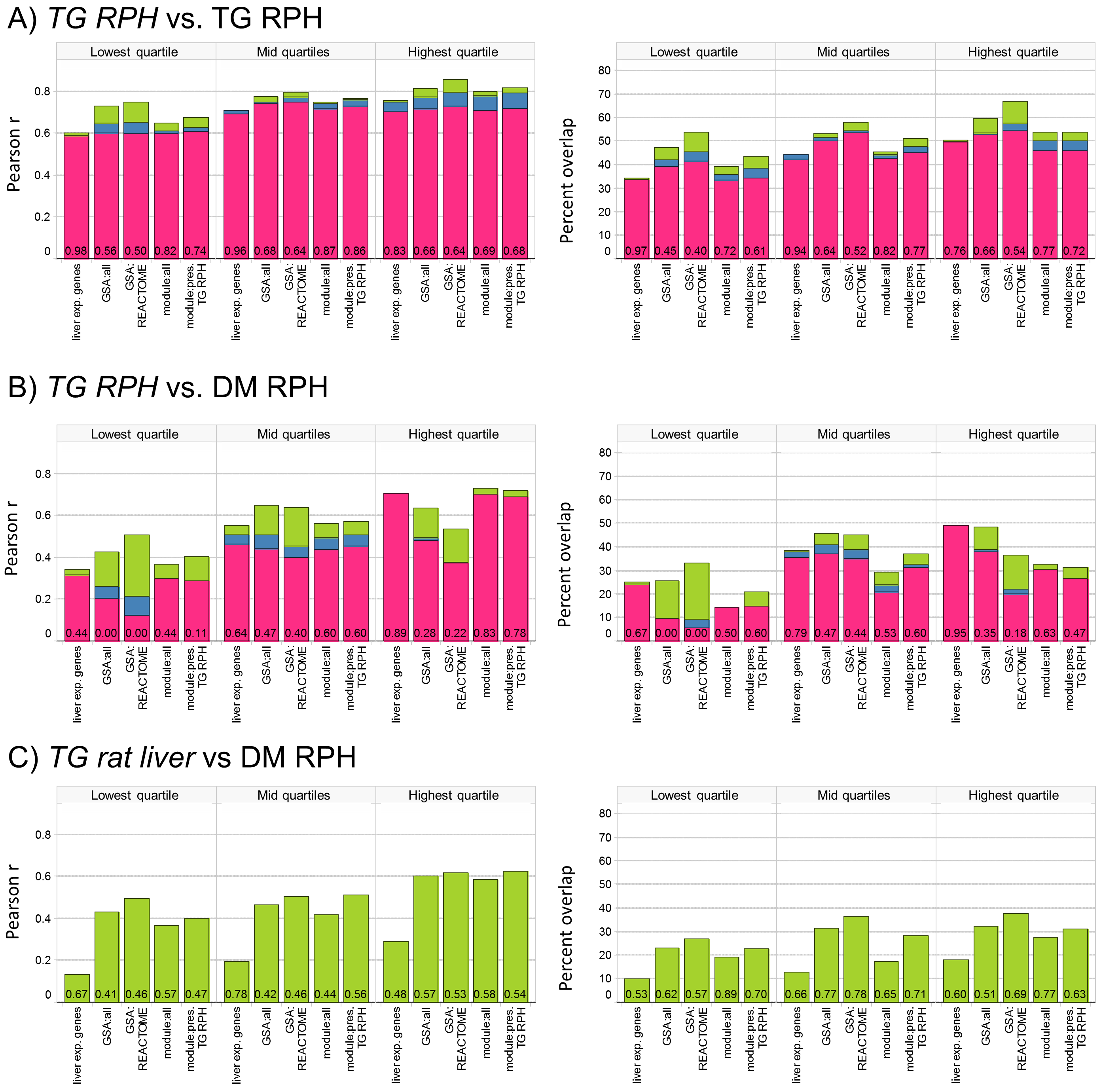

Supplement: S8 Fig — See S4 Fig caption for details, which this figure replicates exactly except for array processing. (TIF) [file pcbi.1004847.s008.tif]

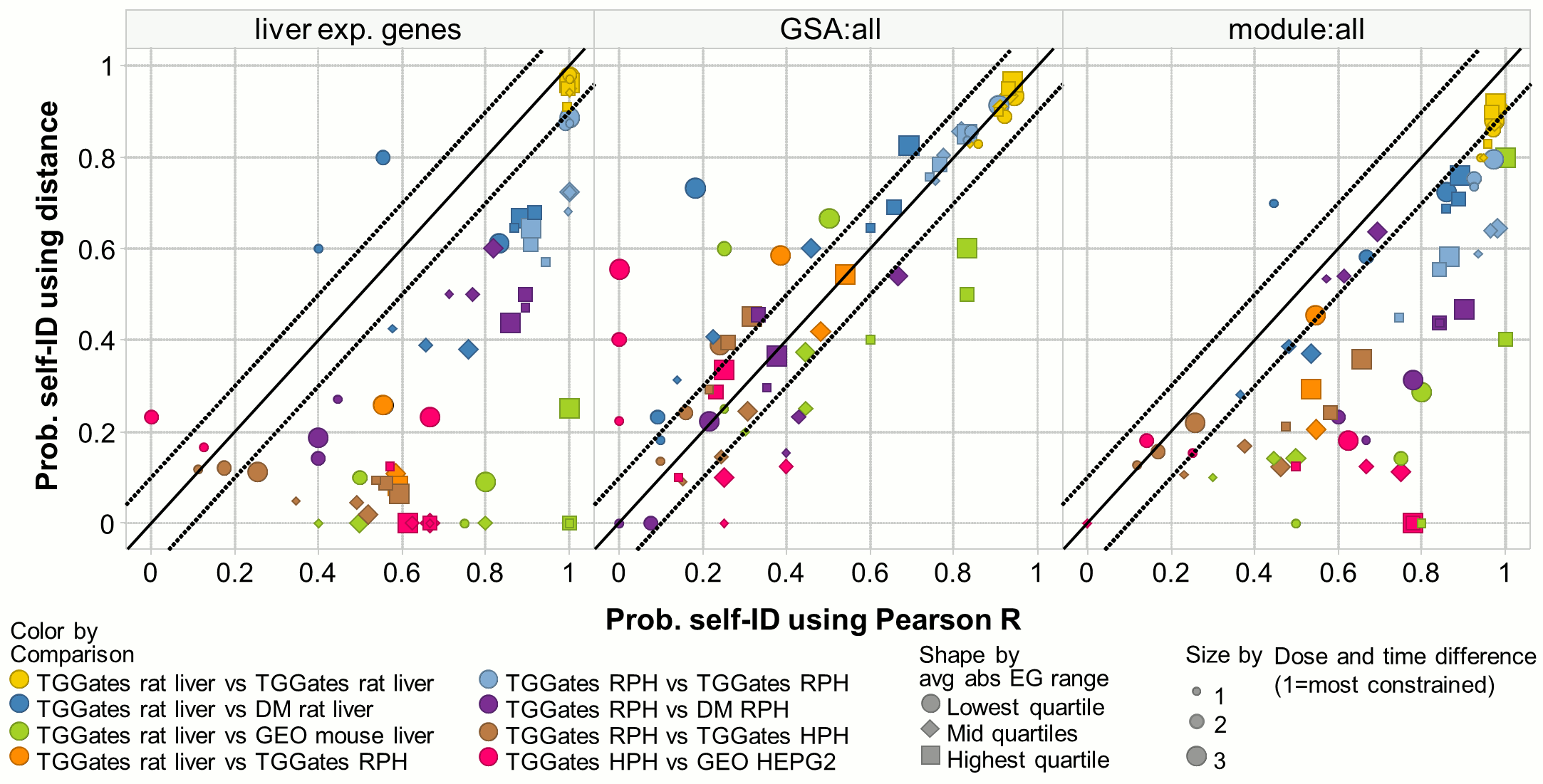

Supplement: S9 Fig — The solid line indicates equal performance, while dashed lines indicate the interval [0.1, -0.1] around the solid line (i.e. an arbitrary definition of “similar performance”). For each axis, increasing self-ID success towards 1 denotes increasing performance. (TIF) [file pcbi.1004847.s009.tif]
