# Supplementary material for: Assessing Concordance of Drug-Induced Transcriptional Response in Rodent Liver and Cultured Hepatocytes
Source: PLoS Comput Biol. 2016 Mar 30;12(3):e1004847. doi: 10.1371/journal.pcbi.1004847 (PMC4814051; doi:10.1371/journal.pcbi.1004847)
Supplement: S1 Table — (DOCX) [file pcbi.1004847.s010.docx]

Table S1. Concordance of TG-GATEs experiment pairs involving the same drug as a function of dose differences, time differences and average absolute eigengene score

| **Comparison** | **Average Pearson R** | | | **Experiment pairs** | | | **Std err Pearson R** | | |
| --- | --- | --- | --- | --- | --- | --- | --- | --- | --- |
|  | **genes** | **GSA** | **modules** | **genes** | **GSA** | **modules** | **genes** | **GSA** | **modules** |
|  | **impact of dose in rat liver** | | | | | | | | |
| repeat dose studies, same time, doses 10 fold apart | 0.47 | 0.46 | 0.46 | 545 | 545 | 545 | 0.0051 | 0.0091 | 0.0087 |
| repeat dose studies, same time, doses 3.3 fold apart | 0.54 | 0.54 | 0.57 | 1040 | 1040 | 1040 | 0.0033 | 0.0059 | 0.0056 |
| single dose studies, same time, doses 10 fold apart | 0.50 | 0.52 | 0.51 | 587 | 587 | 587 | 0.0053 | 0.0085 | 0.0092 |
| single dose studies, same time, doses 3.3 fold apart | 0.56 | 0.56 | 0.62 | 1091 | 1091 | 1091 | 0.0034 | 0.0059 | 0.0058 |
|  | **impact of time in rat liver** | | | | | | | | |
| repeat dose studies, same dose, times 1.9 fold apart | 0.2 | 0.21 | 0.31 | 1264 | 1264 | 1264 | 0.0060 | 0.0081 | 0.0081 |
| repeat dose studies, same dose, times 3.8 fold apart | 0.18 | 0.17 | 0.29 | 839 | 839 | 839 | 0.0067 | 0.0094 | 0.0095 |
| repeat dose studies, same dose, times 7.3 fold apart | 0.15 | 0.16 | 0.25 | 416 | 416 | 416 | 0.0082 | 0.0115 | 0.0124 |
| same dose, 4 day vs 1 day | 0.17 | 0.23 | 0.26 | 361 | 361 | 361 | 0.0091 | 0.0155 | 0.0132 |
| single dose studies, same dose, times 1.5 or 2 fold apart | 0.19 | 0.17 | 0.32 | 913 | 913 | 913 | 0.0065 | 0.0088 | 0.0099 |
| single dose studies, same dose, times 2.67, 3 or 4 fold apart | 0.13 | 0.16 | 0.21 | 913 | 913 | 913 | 0.005 | 0.0082 | 0.0086 |
| single dose studies, same dose, times 4 fold apart | 0.10 | 0.13 | 0.19 | 457 | 457 | 457 | 0.0063 | 0.0123 | 0.0114 |
| single dose studies, same dose, times 8 fold apart | 0.06 | 0.08 | 0.10 | 456 | 456 | 456 | 0.0044 | 0.0113 | 0.0091 |
|  | **impact of avg abs EG in rat liver** | | | | | | | | |
| same time, doses 3.3 fold apart, avg abs EG < 0.26 | 0.50 | 0.49 | 0.49 | 773 | 773 | 773 | 0.0023 | 0.0059 | 0.0048 |
| same time, doses 3.3 fold apart, avg abs EG between 0.26 and 0.36 | 0.55 | 0.55 | 0.61 | 1101 | 1101 | 1101 | 0.0025 | 0.0056 | 0.0049 |
| same time, doses 3.3 fold apart, avg abs EG>0.36 | 0.73 | 0.73 | 0.83 | 257 | 257 | 257 | 0.0081 | 0.0109 | 0.0105 |
|  | **impact of dose in RPH** | | | | | | | | |
| same time, doses 25 fold apart | 0.43 | 0.44 | 0.43 | 378 | 378 | 378 | 0.0056 | 0.0111 | 0.0098 |
| same time, doses 5 fold apart | 0.54 | 0.55 | 0.59 | 732 | 732 | 732 | 0.0037 | 0.0079 | 0.0069 |
|  | **impact of time in RPH** | | | | | | | | |
| same dose, times 3 fold apart | 0.14 | 0.11 | 0.19 | 418 | 418 | 418 | 0.009 | 0.0139 | 0.0126 |
| same dose, times 4 fold apart | 0.04 | 0.04 | 0.05 | 418 | 418 | 418 | 0.0052 | 0.0142 | 0.0123 |
| same dose, times 12 fold apart | 0.02 | 0.03 | 0.00 | 418 | 418 | 418 | 0.0037 | 0.0116 | 0.0103 |
|  | **impact of avg abs EG in RPH** | | | | | | | | |
| same time, doses 5 fold apart, avg abs EG < 0.26 | 0.51 | 0.51 | 0.54 | 546 | 546 | 546 | 0.0033 | 0.0086 | 0.0074 |
| same time, doses 5 fold apart, avg abs EG between 0.26 and 0.36 | 0.57 | 0.61 | 0.67 | 151 | 151 | 151 | 0.0088 | 0.0177 | 0.0150 |
| same time, doses 5 fold apart, avg abs EG > 0.36 | 0.65 | 0.67 | 0.69 | 63 | 63 | 63 | 0.0266 | 0.0330 | 0.0358 |
