# Supplementary material for: Assessing Concordance of Drug-Induced Transcriptional Response in Rodent Liver and Cultured Hepatocytes
Source: PLoS Comput Biol. 2016 Mar 30;12(3):e1004847. doi: 10.1371/journal.pcbi.1004847 (PMC4814051; doi:10.1371/journal.pcbi.1004847)
Supplement: S2 Table — (DOCX) [file pcbi.1004847.s011.docx]

Table S2. Relationship between overall transcriptional activity and concordance of random experiment pairs involving different drugs from TG rat liver

|  | **liver exp. genes** | | **GSA:all** | | **module:all** | |
| --- | --- | --- | --- | --- | --- | --- |
| **avg. abs. EG range^a^** | **median** | **95th percentile** | **median** | **95th percentile** | **median** | **95th percentile** |
|  | **Pearson R** | | | | | |
| <0.2 | 0.01 | 0.10 | 0.03 | 0.30 | 0.03 | 0.23 |
| 0.2-0.3 | 0.02 | 0.11 | 0.02 | 0.35 | 0.03 | 0.30 |
| 0.3-0.4 | 0.03 | 0.16 | 0.03 | 0.41 | 0.07 | 0.40 |
| 0.4-0.5 | 0.06 | 0.25 | 0.08 | 0.47 | 0.14 | 0.50 |
| 0.5-0.6 | 0.11 | 0.31 | 0.12 | 0.51 | 0.23 | 0.57 |
| 0.6-0.8 | 0.14 | 0.38 | 0.12 | 0.60 | 0.27 | 0.67 |
| >0.8 | 0.23 | 0.51 | 0.23 | 0.69 | 0.43 | 0.75 |
|  | **percent overlap** | | | | | |
| <0.2 | 11.9 | 16.3 | 3.3 | 16.3 | 4.8 | 14.3 |
| 0.2-0.3 | 11.9 | 16.6 | 3.3 | 17.4 | 4.8 | 19.1 |
| 0.3-0.4 | 11.9 | 18.8 | 2.2 | 22.8 | 4.8 | 19.1 |
| 0.4-0.5 | 12.4 | 20.5 | 2.2 | 21.7 | 4.8 | 19.1 |
| 0.5-0.6 | 13.0 | 24.5 | 3.3 | 26.1 | 4.8 | 28.6 |
| 0.6-0.8 | 13.5 | 25.8 | 5.4 | 31.5 | 4.8 | 23.8 |
| >0.8 | 16.3 | 31.4 | 8.7 | 40.2 | 4.8 | 28.6 |

^a^ For each pair of experiments, the smallest of two avg. abs. EG values is used; 1000 pairs were sampled at random for each given range of avg. abs. EG; percent overlap medians and 95^th^ percentiles are the same across several ranges because compared to genes, fewer discrete values are possible (i.e. 0/21, 1/21, 2/21, ... for the top 5% of modules, or 21 modules)
