# Supplementary material for: Assessing Concordance of Drug-Induced Transcriptional Response in Rodent Liver and Cultured Hepatocytes
Source: PLoS Comput Biol. 2016 Mar 30;12(3):e1004847. doi: 10.1371/journal.pcbi.1004847 (PMC4814051; doi:10.1371/journal.pcbi.1004847)
Supplement: S3 Table — (DOCX) [file pcbi.1004847.s012.docx]

Table S3. Concordance between rat liver and rat primary hepatocytes (RPH) for select drug classes from TG using co-expression modules preserved in RPH

|  | **Pearson R by quartile of transcriptional activity^a^** | | | |  |
| --- | --- | --- | --- | --- | --- |
| **Class** | **Low** | **Mid** | **High** | **Any** | **Drugs** |
| Antiarrhythmic | 0.36 (21)^b^ | 0.38 (12) | 0.53 (3) | 0.38 (36) | amiodarone, disopyramide, mexiletine, quinidine |
| Anticonvulsants | 0.32 (26) | 0.41 (8) | NS | 0.36 (36) | carbamazepine, phenytoin, trimethadione, valproic acid |
| H1 antagonists | 0.39 (21) | 0.44 (14) | NS^c^ | 0.41 (36) | dexchlorpheniramine, hydroxyzine, methapyrilene, promethazine |
| H2 antagonists | 0.33 (22) | 0.38 (5) | NS | 0.34 (27) | cimetidine, famotidine, ranitidine |
| NSAIDs | 0.36 (70) | 0.39 (27) | 0.55 (11) | 0.39 (108) | aspirin, bendazac, diclofenac, ibuprofen, indomethacin, lornoxicam, mefenamic acid, meloxicam, naproxen, nimesulide, sulfasalazine, sulindac |
| Phenothiazines | 0.33 (21) | 0.3 (5) | NS | 0.32 (27) | chlorpromazine, fluphenazine, thioridazine |
| PPAR alpha agonists | 0.38 (29) | 0.49 (6) | NS | 0.4 (36) | clofibrate, fenofibrate, gemfibrozil, pirinixic acid |
| Tricyclic antidepressants | 0.39 (21) | 0.44 (14) | NS | 0.41 (36) | amitriptyline, clomipramine, doxepin, imipramine |

^a^ Data from Figure 4A by drug class for the subset of 207 modules preserved in TG RPH. ^b^ The number of experiment pairs used for calculating average Pearson R. ^c^ Not shown, because fewer than 3 drug pairs contribute to the average. Abbreviations: non-steroidal anti-inflammatory drugs (NSAIDs), histamine receptor H1 (H1), histamine receptor H2 (H2), peroxisome proliferator-activated receptor (PPAR)
