# Supplementary material for: Assessing Concordance of Drug-Induced Transcriptional Response in Rodent Liver and Cultured Hepatocytes
Source: PLoS Comput Biol. 2016 Mar 30;12(3):e1004847. doi: 10.1371/journal.pcbi.1004847 (PMC4814051; doi:10.1371/journal.pcbi.1004847)
Supplement: S4 Table — (DOCX) [file pcbi.1004847.s013.docx]

Table S4. Concordance of LC_50_ values obtained by viability assays using cultured rat primary hepatocytes and human HepG2 cells

|  | **Number of Lilly compounds where cytotox LC50s**  **are within 5 fold or agree by qualifier*** | |
| --- | --- | --- |
| **Result category** | **Yes** | **No** |
| Both LC_50_s non-qualified | 509 | 26 |
| HepG2 LC_50_ > 100 uM* | 224 | 14 |
| RPH LC_50_ > 100 uM* | 193 | 23 |
| Both LC_50_s > 100 uM | 1694 | 0 |

Cytotoxicity in RPH is assessed via lactate dehydrogenase (LDH) leakage from cells. Compounds are incubated at 100, 80, 60, 40, 20, 10, and 1 μM final concentration, and % LDH release normalized using DMSO as negative control (0% release) and 100 µM chlorpromazine as positive control (100% release). Normalized LDH levels are curve fit, with the LC_50_ indicating the concentration that causes 50% cytolethality compared to the positive control. Cytotoxicity in HepG2 is determined by nuclear count using Hoechst staining, using amiodarone as positive control. Compounds are incubated at 100,80,60,40,30,20,10,5,2,1 μM, and % toxicity calculated as described for RPH. * A qualified LC50 is a result with prefix ‘>’, i.e. no cytotoxicity within range of concentration studied (1 uM to 100 uM). “Agreement by qualifier” is illustrated with the following: when HepG2 LC_50_ > 100 uM and RPH LC_50_ = 20.1 uM or greater, the results are counted as within 5 fold
