# Supplementary material for: Assessing Concordance of Drug-Induced Transcriptional Response in Rodent Liver and Cultured Hepatocytes
Source: PLoS Comput Biol. 2016 Mar 30;12(3):e1004847. doi: 10.1371/journal.pcbi.1004847 (PMC4814051; doi:10.1371/journal.pcbi.1004847)
Supplement: S5 Table — (DOCX) [file pcbi.1004847.s014.docx]

Table S5. Concordance of mouse primary hepatocytes vs. rat primary hepatocytes and mouse liver

|  | | | **Pearson R** | | | | **Percent overlap** | | |
| --- | --- | --- | --- | --- | --- | --- | --- | --- | --- |
| **Drug** | **Doses MPH / RPH (**µ**M)^c^** | **avg abs EG range^e^** | **liver exp. genes** | **GSA:all** | **Modules:all** | **liver exp. genes** | | **GSA:all** | **Modules:all** |
|  | **RPH vs MPH^a^** | | | | | | | | |
| 2-acetylaminofluorene | 125 / 50 | Low | 0.07 (1)^d^ | -0.06 (1) | 0.26 (1) | 11 (2) | | 10 (2) | 0 (2) |
| cisplatin | 7 / 8 | High | 0.43 (1) | 0.32 (1) | 0.58 (1) | 29 (1) | | 16 (1) | 28 (1) |
| cyclosporin A | 1 / 1 | High | 0.23 (1) | 0.31 (1) | 0.56 (1) | 16 (1) | | 22 (2) | 38 (1) |
| phenacetin | 1500 / 600 | Low | 0.02 (2) | 0.18 (2) | 0.02 (2) | 9 (1) | | 4 (1) | 0 (1) |
| pirinixic acid | 300 / 200 | Low | 0.12 (2) | 0.36 (2) | 0.16 (2) | 9 (2) | | 20 (2) | 0 (1) |
|  |  |  |  |  |  |  | |  |  |
|  | **Mouse liver vs MPH^b^** | | | | | | | | |
| 2-acetylaminofluorene |  | Mid | 0.04 (2) | 0.12 (1) | 0.12 (1) | 12 (1) | | 1 (2) | 10 (1) |
| aflatoxin B1 |  | Mid | 0.07 (2) | 0.22 (1) | 0.18 (2) | 9 (2) | | 10 (1) | 10 (2) |
| benzopyrene |  | Low | 0.07 (2) | 0.05 (1) | 0.14 (2) | 8 (2) | | 7 (2) | 5 (2) |
| cisplatin |  | Mid | -0.02 (2) | -0.23 (1) | -0.19 (1) | 5 (2) | | 2 (2) | 5 (2) |
| cyclosporin A |  | Mid | 0.13 (2) | 0.25 (2) | 0.34 (2) | 9 (1) | | 13 (2) | 43 (1) |
| N nitrosodimethylamine |  | Mid | 0.23 (2) | 0.49 (2) | 0.44 (2) | 13 (2) | | 15 (2) | 14 (2) |
| phenacetin |  | Low | 0.06 (2) | 0.25 (1) | 0.29 (2) | 12 (2) | | 9 (2) | 10 (1) |
| pirinixic acid |  | High | 0.22 (1) | 0.52 (1) | 0.45 (1) | 15 (1) | | 32 (2) | 19 (1) |

Comparing profiles for drugs at single concentration and following 1 or 2 days of exposure in mouse primary hepatocytes from E-MEXP-2539, E-MEXP-2209, E-MEXP-2636 in EBI ArrayExpress and GSE57129 in NCBI GEO to ^a^ profiles from TG RPH with 1 day incubation and ^b^ profiles from GEO male mouse liver following 7 days of exposure (GSE43977) except N-nitrosodimethylamine (15 days) or pirinixic acid (4 days); 4 and 15 day data are from GSE44783, and the time point which maximizes concordance vs. MPH was selected. A direct comparison to rat liver vs. RPH in TG is made difficult by the much more limited range of doses and times available in the mouse and therefore greater possible influence of pharmacokinetics in explaining low concordance. ^c^ For comparisons to RPH, the most similar dose was selected and for all cases except phenacetin this is also the dose giving the highest concordance vs. MPH; the 24 µM dose for phenacetin gave marginally higher concordance than the 600 µM dose. ^d^ the MPH exposure in days giving highest concordance vs. RPH or mouse liver is selected and shown in parentheses. ^e^ The range for avg. abs. eigengene into which the treatment falls, as defined in Figures 2 and 4. Some drugs are available in 2 or more of the above data series, and the profile which maximizes concordance vs. RPH or mouse liver was selected.
