# Supplementary material for: Assessing Concordance of Drug-Induced Transcriptional Response in Rodent Liver and Cultured Hepatocytes
Source: PLoS Comput Biol. 2016 Mar 30;12(3):e1004847. doi: 10.1371/journal.pcbi.1004847 (PMC4814051; doi:10.1371/journal.pcbi.1004847)
Supplement: S6 Table — (DOCX) [file pcbi.1004847.s015.docx]

Table S6. Pearson correlation between expression levels of genes in control samples for MAS5 and RMA normalized intensities

|  |  | All genes | | | 9071 rat liver-expressed genes | | |
| --- | --- | --- | --- | --- | --- | --- | --- |
| Source 1 | Source 2 | R- MAS5 | R-RMA | # genes^a^ | R- MAS5 | R-RMA | # genes^b^ |
| DM rat liver | TG rat liver | 0.994 | 0.993 | 14078 | 0.987 | 0.987 | 9071 |
| DM rat liver | GEO mouse liver | 0.829 | 0.835 | 12556 | 0.750 | 0.755 | 8402 |
| DM rat liver | GEO human liver | 0.752 | 0.747 | 12501 | 0.662 | 0.664 | 8348 |
| DM rat liver | DM RPH | 0.900 | 0.888 | 14078 | 0.751 | 0.769 | 9071 |
| DM rat liver | TG RPH | 0.908 | 0.894 | 14078 | 0.772 | 0.784 | 9071 |
| DM rat liver | GEO MPH | 0.765 | 0.760 | 12556 | 0.608 | 0.623 | 8402 |
| DM rat liver | TG HPH | 0.704 | 0.685 | 12501 | 0.502 | 0.508 | 8349 |
| DM rat liver | GEO HepG2 | 0.629 | 0.606 | 12501 | 0.397 | 0.411 | 8348 |
| TG rat liver | GEO mouse liver | 0.825 | 0.830 | 12556 | 0.744 | 0.751 | 8402 |
| TG rat liver | GEO human liver | 0.745 | 0.742 | 12501 | 0.651 | 0.657 | 8348 |
| TG rat liver | DM RPH | 0.896 | 0.882 | 14078 | 0.747 | 0.762 | 9071 |
| TG rat liver | TG RPH | 0.910 | 0.895 | 14078 | 0.779 | 0.790 | 9071 |
| TG rat liver | GEO MPH | 0.763 | 0.757 | 12556 | 0.610 | 0.625 | 8402 |
| TG rat liver | TG HPH | 0.699 | 0.681 | 12501 | 0.500 | 0.507 | 8349 |
| TG rat liver | GEO HepG2 | 0.623 | 0.601 | 12501 | 0.395 | 0.409 | 8348 |
| GEO mouse liver | GEO human liver | 0.768 | 0.757 | 14898 | 0.660 | 0.657 | 8139 |
| GEO mouse liver | DM RPH | 0.780 | 0.776 | 12556 | 0.632 | 0.634 | 8402 |
| GEO mouse liver | TG RPH | 0.774 | 0.771 | 12556 | 0.631 | 0.632 | 8402 |
| GEO mouse liver | GEO MPH | 0.913 | 0.903 | 15691 | 0.836 | 0.831 | 8402 |
| GEO mouse liver | TG HPH | 0.732 | 0.717 | 14898 | 0.559 | 0.550 | 8140 |
| GEO mouse liver | GEO HepG2 | 0.663 | 0.640 | 14898 | 0.459 | 0.449 | 8139 |
| GEO human liver | DM RPH | 0.710 | 0.700 | 12501 | 0.567 | 0.572 | 8348 |
| GEO human liver | TG RPH | 0.712 | 0.702 | 12501 | 0.580 | 0.582 | 8348 |
| GEO human liver | GEO MPH | 0.715 | 0.702 | 14898 | 0.550 | 0.557 | 8139 |
| GEO human liver | TG HPH | 0.870 | 0.847 | 15991 | 0.771 | 0.752 | 8348 |
| GEO human liver | GEO HepG2 | 0.770 | 0.746 | 15991 | 0.622 | 0.612 | 8348 |
| DM RPH | TG RPH | 0.981 | 0.980 | 14078 | 0.961 | 0.963 | 9071 |
| DM RPH | GEO MPH | 0.812 | 0.806 | 12556 | 0.712 | 0.708 | 8402 |
| DM RPH | TG HPH | 0.777 | 0.771 | 12501 | 0.671 | 0.673 | 8349 |
| DM RPH | GEO HepG2 | 0.703 | 0.688 | 12501 | 0.568 | 0.570 | 8348 |
| TG RPH | GEO MPH | 0.800 | 0.798 | 12556 | 0.698 | 0.698 | 8402 |
| TG RPH | TG HPH | 0.772 | 0.769 | 12501 | 0.668 | 0.673 | 8349 |
| TG RPH | GEO HepG2 | 0.693 | 0.681 | 12501 | 0.554 | 0.560 | 8348 |
| GEO MPH | TG HPH | 0.760 | 0.745 | 14898 | 0.626 | 0.616 | 8140 |
| GEO MPH | GEO HepG2 | 0.697 | 0.673 | 14898 | 0.534 | 0.520 | 8139 |
| TG HPH | GEO HepG2 | 0.876 | 0.862 | 15991 | 0.813 | 0.805 | 8348 |

Correlation of gene-level log-intensities at baseline (untreated samples) across systems and sources. ^a^ number of genes used to calculate correlation; values differ across systems due to missing ortholog mappings or absence of a given gene on a microarray (see methods on mapping of probe sets to genes and ortholog selection). ^b^ number of genes when selecting only from the subset of 9071 rat liver expressed genes. While the trend is similar to results from all genes, lower R values are obtained because the range of expression is truncated in the middle of the full range of intensities observed, which tends to be the ‘fattest’ part of the distribution in expression correlation analysis.
