# Supplementary material for: Assessing Concordance of Drug-Induced Transcriptional Response in Rodent Liver and Cultured Hepatocytes
Source: PLoS Comput Biol. 2016 Mar 30;12(3):e1004847. doi: 10.1371/journal.pcbi.1004847 (PMC4814051; doi:10.1371/journal.pcbi.1004847)
Supplement: S7 Table — (DOCX) [file pcbi.1004847.s016.docx]

Table S7. Summary of clinical chemistry and histology findings for 29 day methapyrilene and 1 day bortezomib treated rats

|  | 29 day repeat-dose methapyrilene rodent study | | |
| --- | --- | --- | --- |
| Dose | 10 mg/kg | 30 mg/kg | 100 mg/kg |
|  |  |  |  |
|  | Clinical chemistry (% change from control group) | | |
| ALB | 5 | -5 | -33 |
| ALP | 21 | 21 | 82 |
| ALT | -1 | 81 | 128 |
| AST | 3 | 104 | 528 |
| Chol | 7 | 35 | -38 |
| GGT | 0 | 2 | 417 |
| Glu | -2 | -8 | -37 |
| T Bili | 0 | 0 | 911 |
|  |  |  |  |
|  | Liver histology grades (number of animals) | | |
| Single cell necrosis | normal (3) | normal (1), slight (2) | slight (3) |
| Biliary hyperplasia | normal (3) | normal (3) | moderate (3) |
| Hypertrophy | normal (3) | slight (3) | slight (3) |
| Inflammation | normal (3) | normal (1), slight (2) | slight (3) |
| Increased mitosis | normal (3) | normal (1), slight (2) | slight (3) |
|  |  |  |  |
|  | 1 day single-dose bortezomib rodent study | | |
| Dose | 0.1 mg/kg | 0.3 mg/kg | 1 mg/kg |
|  |  |  |  |
|  | Clinical chemistry (% change from control group) | | |
| ALB | 3 | -13 | -34 |
| ALP | -4 | -4 | -29 |
| ALT | 4 | 44 | 288 |
| AST | 0 | 2 | 396 |
| Chol | 9 | 14 | -45 |
| GGT | 0 | 0 | 62 |
| Glu | -1 | 1 | -26 |
| T Bili | 0 | 0 | 504 |
|  |  |  |  |
|  | Liver histology grades (number of animals) | | |
| Single cell necrosis | normal (3) | normal (3) | slight (1), moderate (2) |
| Necrosis | normal (3) | normal (3) | normal (1), slight (1), moderate (1) |
| Hypertrophy | normal (3) | normal (1), slight (1), moderate (1) | marked (3) |
| Vacuolation | normal (3) | normal (2), slight (1) | moderate (1), marked (2) |
