# Supplementary material for: Assessing Concordance of Drug-Induced Transcriptional Response in Rodent Liver and Cultured Hepatocytes
Source: PLoS Comput Biol. 2016 Mar 30;12(3):e1004847. doi: 10.1371/journal.pcbi.1004847 (PMC4814051; doi:10.1371/journal.pcbi.1004847)
Supplement: S8 Table — (DOCX) [file pcbi.1004847.s017.docx]

Table S8. Re-analysis of MAQC-I Toxicogenomics results in rat liver for three treatments and comparison to TG rat liver within-source concordance

| **drug (min avg. abs. EG for both sites^a^)** | **method** | **Between sites R** | **Between sites percent overlap** | **Std dev distance for R^b^** | **Std dev distance for overlap^b^** |
| --- | --- | --- | --- | --- | --- |
| aristolochic acid (0.40) | liver exp. genes | 0.78 | 61 | 2.6 | 3.4 |
|  | GSA:all | 0.35 | 32 | -1.3 | 0.1 |
|  | GSA:REACTOME | 0.27 | 40 | -1.4 | 0.2 |
|  | module:all | 0.87 | 67 | 1.5 | 2.1 |
|  | module:pres. TG-liver | 0.92 | 82 | 1.5 | 2.2 |
| riddelliine (0.57) | liver exp. genes | 0.91 | 72 | 1.5 | 1.7 |
|  | GSA:all | 0.62 | 42 | -0.8 | -0.3 |
|  | GSA:REACTOME | 0.77 | 50 | 0.0 | 0.0 |
|  | module:all | 0.97 | 67 | 0.9 | 0.6 |
|  | module:pres. TG-liver | 0.99 | 82 | 0.9 | 1.0 |
| comfrey (1.14) | liver exp. genes | 0.93 | 79 | 1.6 | 2.2 |
|  | GSA:all | 0.96 | 82 | 1.4 | 1.8 |
|  | GSA:REACTOME | 0.95 | 80 | 1.0 | 1.2 |
|  | module:all | 0.98 | 86 | 0.9 | 1.6 |
|  | module:pres. TG-liver | 0.99 | 100 | 0.9 | 1.8 |

Results for GSE5350 were re-analyzed starting from CEL files for the two Affymetrix sites included in the MAQC-I toxicogenomics project. ^a^ Smallest value of the avg. abs. EG score for results from the two sites. ^b^ The difference between Pearson R or overlap metric for the MAQC study vs. the TG rat liver within-source concordance at high stringency (same time, doses within 3 fold), measured in standard deviations for the TG rat liver results in the same quartile of transcriptional activity. A value of +1 indicates that the MAQC result for the given drug is 1 standard deviation above the average TG rat liver result; a value of -1 indicates a concordance 1 standard deviation below. As such, only aristolochic acid is atypical, where concordance is much higher than results reported here for the TG rat liver within-source concordance. It should be noted that the same sample aliquots were distributed to various sites for microarray analysis in MAQC, whereas the TG rat liver comparison involves distinct samples from rats treated with different doses.
