# Supplementary material for: Assessing Concordance of Drug-Induced Transcriptional Response in Rodent Liver and Cultured Hepatocytes
Source: PLoS Comput Biol. 2016 Mar 30;12(3):e1004847. doi: 10.1371/journal.pcbi.1004847 (PMC4814051; doi:10.1371/journal.pcbi.1004847)
Supplement: S9 Table — (DOCX) [file pcbi.1004847.s018.docx]

Table S9. Probability of success for self-identification when comparing expression profiles of drugs across models and sources

| **Comparison** | **liver exp. genes** | **GSA:all** | **GSA: REACTOME** | **module:all** |
| --- | --- | --- | --- | --- |
|  | **Pearson R** | | | |
| *TGGates rat liver* vs TGGates rat liver | 1.00 | 0.94 | 0.89 | 0.98 |
| *TGGates rat liver* vs DM rat liver | 0.82 | 0.58 | 0.69 | 0.71 |
| *TGGates rat liver* vs GEO mouse liver | 0.75 | 0.64 | 0.75 | 0.75 |
| *TGGates rat liver* vs TGGates RPH | 0.59 | 0.51 | 0.50 | 0.54 |
| *TGGates rat liver* vs DM RPH | 0.47 | 0.35 | 0.43 | 0.59 |
| *TGGates RPH* vs TGGates RPH | 0.95 | 0.83 | 0.73 | 0.92 |
| *TGGates RPH* vs DM RPH | 0.84 | 0.52 | 0.37 | 0.80 |
| *TGGates RPH* vs TGGates HPH | 0.56 | 0.31 | 0.31 | 0.56 |
| *TGGates HPH* vs GEO HEPG2 | 0.64 | 0.25 | 0.37 | 0.76 |
|  |  |  |  |  |
|  | **Percent overlap** | | | |
| *TGGates rat liver* vs TGGates rat liver | 1.00 | 0.91 | 0.84 | 0.97 |
| *TGGates rat liver* vs DM rat liver | 0.75 | 0.67 | 0.59 | 0.93 |
| *TGGates rat liver* vs GEO mouse liver | 0.75 | 0.75 | 0.67 | 0.88 |
| *TGGates rat liver* vs TGGates RPH | 0.57 | 0.56 | 0.61 | 0.76 |
| *TGGates rat liver* vs DM RPH | 0.54 | 0.56 | 0.61 | 0.65 |
| *TGGates RPH* vs TGGates RPH | 0.93 | 0.56 | 0.56 | 0.88 |
| *TGGates RPH* vs DM RPH | 0.87 | 0.57 | 0.41 | 0.70 |
| *TGGates RPH* vs TGGates HPH | 0.31 | 0.32 | 0.38 | 0.67 |
| *TGGates HPH* vs GEO HEPG2 | 0.58 | 0.33 | 0.43 | 0.83 |

Each experiment is compared to profiles in reference system (italicized, left of “vs”) and the most similar reference profile for the same drug is selected with no constraint on dose or time differences. The probability that the level of concordance for the most similar profile exceeds the concordance of random pairs involving different drugs at p < 0.05 is reported, as a simple average over the mid and high avg. abs. EG ranges (methods).
