# Supplementary material for: Assessing Concordance of Drug-Induced Transcriptional Response in Rodent Liver and Cultured Hepatocytes
Source: PLoS Comput Biol. 2016 Mar 30;12(3):e1004847. doi: 10.1371/journal.pcbi.1004847 (PMC4814051; doi:10.1371/journal.pcbi.1004847)
Supplement: S10 Table — (DOCX) [file pcbi.1004847.s019.docx]

Table S10. Comparing concordance and probability of success in self-identification within TG-GATEs data for legacy and modern Affymetrix array processing

|  | **Concordance** | | | | **Prob. self-ID success** | | | |
| --- | --- | --- | --- | --- | --- | --- | --- | --- |
|  | **Pearson R** | | **Percent overlap** | | **Pearson R** | | **Percent overlap** | |
| **Method** | **L^a^** | **M^a^** | **L** | **M** | **L** | **M** | **L** | **M** |
|  | **TG rat liver vs. TG rat liver^b^** | | | | | | | |
| genes | 0.57 | 0.61 | 40 | 44 | 1.00 | 1.00 | 1.00 | 0.99 |
| GSA | 0.58 | 0.63 | 30 | 40 | 0.86 | 0.73 | 0.77 | 0.67 |
| modules | 0.63 | 0.64 | 35 | 35 | 0.95 | 0.91 | 0.84 | 0.81 |
|  |  |  |  |  |  |  |  |  |
|  | **TG RPH vs. TG RPH^b^** | | | | | | | |
| genes | 0.54 | 0.62 | 39 | 37 | 0.99 | 0.96 | 0.98 | 0.95 |
| GSA | 0.57 | 0.64 | 31 | 43 | 0.81 | 0.60 | 0.63 | 0.51 |
| modules | 0.61 | 0.63 | 34 | 36 | 0.91 | 0.82 | 0.78 | 0.75 |
|  |  |  |  |  |  |  |  |  |
|  | **TG rat liver vs. TG RPH^c^** | | | | | | | |
| genes | 0.09 | 0.15 | 12 | 11 | 0.57 | 0.55 | 0.60 | 0.47 |
| GSA | 0.36 | 0.52 | 20 | 29 | 0.43 | 0.50 | 0.52 | 0.60 |
| modules | 0.30 | 0.34 | 19 | 20 | 0.55 | 0.55 | 0.78 | 0.77 |

^a^ L denotes legacy processing of array data, M denotes modern. Legacy uses manufacturer’s probe sets and MAS5; modern uses updated probe sets (i.e. “Brainarray” probe sets) [[53](#_ENREF_53)] with RMA normalization. ^b^For rat liver and RPH, only pairs of experiments for the same time point and similar doses (≤3.3 fold rat liver and ≤5 fold for RPH) are considered. ^c^For rat liver vs. RPH, each RPH experiment is matched to the most concordant rat liver experiment for the same compound (i.e. level 3 comparisons from main text).
