# Supplementary material for: Assessing Concordance of Drug-Induced Transcriptional Response in Rodent Liver and Cultured Hepatocytes
Source: PLoS Comput Biol. 2016 Mar 30;12(3):e1004847. doi: 10.1371/journal.pcbi.1004847 (PMC4814051; doi:10.1371/journal.pcbi.1004847)
Supplement: S11 Table — (DOCX) [file pcbi.1004847.s020.docx]

Table S11. Concordance of module scores using gene variability scaling and module weight calibration from DM rat liver vs. scores obtained by rescaling or recalibrating modules in other systems

| Scaling | Weights | Experiments used for comparison | subset of modules | min | 10th percentile | median |
| --- | --- | --- | --- | --- | --- | --- |
| TG liver | TG liver | TG liver | 415 (all) | 0.88 | 0.93 | 0.96 |
| TG liver | TG liver | TG liver | 344 (Z-sum ≥ 3 in TG liver) | 0.88 | 0.94 | 0.96 |
| DM RPH | DM liver | DM RPH | 415 (all) | 0.92 | 0.94 | 0.96 |
| DM RPH | DM liver | TG RPH | 415 (all) | 0.92 | 0.94 | 0.96 |
| DM RPH | DM RPH | DM RPH | 415 (all) | 0.68 | 0.76 | 0.81 |
| DM RPH | DM RPH | TG RPH | 415 (all) | 0.52 | 0.70 | 0.77 |
| DM RPH | DM RPH | DM RPH | 154 (Z-sum ≥ 3 in DM RPH) | 0.78 | 0.86 | 0.89 |
| DM RPH | DM RPH | TG RPH | 154 (Z-sum ≥ 3 in DM RPH) | 0.65 | 0.80 | 0.87 |
| TG RPH | TG RPH | DM RPH | 415 (all) | 0.73 | 0.81 | 0.86 |
| TG RPH | TG RPH | TG RPH | 415 (all) | 0.62 | 0.74 | 0.81 |
| TG RPH | TG RPH | DM RPH | 207 (Z-sum ≥ 3 in TG RPH) | 0.82 | 0.88 | 0.91 |
| TG RPH | TG RPH | TG RPH | 207 (Z-sum ≥ 3 in TG RPH) | 0.70 | 0.84 | 0.89 |
| TG HPH | DM liver | TG HPH | 415 (all) | 0.65 | 0.78 | 0.83 |
| TG HPH | TG HPH | TG HPH | 415 (all) | 0.23 | 0.37 | 0.48 |
| TG HPH | TG HPH | TG HPH | 97 (Z-sum ≥ 2 in TG HPH) | 0.00 | 0.44 | 0.65 |
| TG HPH | TG HPH | TG HPH | 56 (Z-sum ≥ 3 in TG HPH) | 0.04 | 0.43 | 0.70 |
