# Supplementary material for: Assessing Concordance of Drug-Induced Transcriptional Response in Rodent Liver and Cultured Hepatocytes
Source: PLoS Comput Biol. 2016 Mar 30;12(3):e1004847. doi: 10.1371/journal.pcbi.1004847 (PMC4814051; doi:10.1371/journal.pcbi.1004847)
Supplement: S12 Table — (DOCX) [file pcbi.1004847.s021.docx]

Table S12. Spearman rho correlation of logFC standard deviation for 9071 liver-expressed genes (8349 for HPH due to missing orthologs) across various systems

|  | DM rat liver | TG rat liver | DM RPH | TG RPH | TG HPH |
| --- | --- | --- | --- | --- | --- |
| DM rat liver | − | 0.87 | 0.70 | 0.69 | 0.50 |
| TG rat liver | 0.87 | − | 0.65 | 0.75 | 0.55 |
| DM RPH | 0.70 | 0.65 | − | 0.84 | 0.60 |
| TG RPH | 0.69 | 0.75 | 0.84 | − | 0.67 |
| TG HPH | 0.50 | 0.55 | 0.60 | 0.67 | − |
